# Development and validation of a measurement instrument for student assessment of quality physical education in Chinese secondary schools

**DOI:** 10.1371/journal.pone.0324227

**Published:** 2025-06-05

**Authors:** Ling Qin, Walter King Yan Ho, Selina Khoo

**Affiliations:** 1 Faculty of Physical Education and Health Science, Chongqing Normal University, Chongqing, China; 2 Faculty of Sports and Exercise Science, Universiti Malaya, Kuala Lumpur, Malaysia; 3 Tokyo Gakugei University, Tokyo, Japan; Southwest University, CHINA

## Abstract

There is a growing emphasis on developing Quality Physical Education (QPE) programs. However, a research gap exists due to the lack of measurement instruments to assess QPE for students. This study aimed to develop an instrument to assess QPE implementation and its validity and reliability in China. This study comprised three phases. In Phase 1, a conceptual framework for QPE was established using grounded theory based on interviews with 22 PE teachers and 20 students. In Phase 2, using the conceptual framework from Phase 1, we developed an item pool supported by a literature review, expert evaluations, and student interviews. In Phase 3, a cross-sectional study was conducted with secondary school students (705 participants) to analyse the items and assess the instrument’s reliability and validity through exploratory and confirmatory factor analysis, as well as test-retest analysis. The final 45-item instrument, comprising four subscales (student, family, school, and community) across 10 factors, demonstrated strong validity and reliability. Model fit indices met established thresholds (e.g., CFI and TLI ≥ .90, as well as RMSEA ≤ .08). The composite reliability and average variance extracted values for each factor exceeded.7 and.5, respectively, with the test-retest reliability also exceeding.7, indicating high reliability and validity. This study addresses a critical methodological gap in QPE research by developing a culturally contextualized assessment instrument that explicitly identifies and measures students’ perceptions of QPE implementation. This instrument enables systematic monitoring of QPE practices from students’ perspectives, informing evidence-based policymaking and resource allocation. Integrating student, family, school, and community subscales supports holistic interventions to improve the quality of PE.

## Introduction

Although the benefits of physical activity (PA) are well-documented [1–4], global data indicate that only 27% to 33% of children and adolescents meet the recommended 60 minutes of moderate to vigorous PA each day [5]. In response, quality physical education (QPE) programs have been proposed to improve students’ PA and foster healthier lifestyles and initially introduced by the National Association for Sport and Physical Education [6]. The United Nations Educational, Scientific and Cultural Organization has established a general definition of QPE as (p. 9) ‘the planned, progressive, inclusive learning experience that forms part of the curriculum in early years, primary, and secondary education’ [7]. This definition emphasizes inclusivity, flexibility, progression, and collaborative efforts among schools, communities, and governments to promote PA [8]. Studies also point to QPE’s benefits in enhancing PA levels. Masurier and Corbin [9] outlined 10 reasons for QPE in schools, such as its ability to provide unique opportunities for PA, promote lifelong physical fitness and wellness, and educate the total person. Similarly, Dudley et al. [10] contended that a well-implemented QPE program can significantly benefit students by increasing PA levels. Moreover, QPE programs are designed to cultivate positive attitudes toward PA by promoting the enjoyment of movement and the development of transferable physical skills [11]. To effectively achieve QPE, Ho et al. [12] have emphasised the importance of reliable and valid instruments to identify and measure QPE practice.

Reliable instruments remain underdeveloped, as demonstrated by two notable studies. Ho et al. [12] developed a questionnaire to examine professionals’ perception of the quality of PE in school settings. This questionnaire consisted of 48 items across eight dimensions: skill development and bodily awareness, facilities and norms in PE, quality teaching of PE, plans for feasibility and accessibility of PE, social norms and cultural practice, governmental input for PE, cognitive skills development, and habituated behaviour in physical activities. He et al. [13] developed a questionnaire to assess the implementation quality of PE programs in Chinese junior secondary schools. They conducted an exploratory factor analysis (EFA) to identify the factor structure and relevant items. The study concluded with a questionnaire comprising five dimensions and 38 items, covering organization and management, education and teaching, conditions guarantee, student physical fitness, as well as supervision and inspection.

However, these studies have limitations. First, He et al. [13] based their questionnaire on a single Chinese policy from 2014 [14], which may not fully capture the broader or most current context developments. Second, the study relied only on EFA without performing confirmatory factor analysis (CFA) to assess the instrument’s construct validity, which deviates from the standard scale development process [15]. Finally, the studies by Ho et al. [12] and He et al. [13] focused on PE teachers or professionals, neglecting the vital perspective of students. It is crucial to survey students, as their learning environment, teaching methods, and curriculum significantly influence their academic performance, engagement, and overall satisfaction [16–18]. Moreover, Mercier et al. [19] found that students’ attitudes towards PE was positively associated with their PA levels and physical fitness. Therefore, investigating students’ experiences with QPE is essential for analysing students’ attitudes and experiences in PE, informing interventions for lifelong PA, and guiding improvements for future PE quality.

In addition, QPE faces definitional challenges across fields such as education, psychology, and public health sciences [10], which complicates the development of reliable measurement instruments. Clear conceptual definitions are essential for ensuring the validity and reliability of such instruments [15]. The United Nations Educational, Scientific and Cultural Organization has expanded the understanding of QPE, promoting a broader, more holistic perspective [8]. This expanded definition has influenced national education systems, resulting in differing approaches to QPE based on local contexts [20–22]. Therefore, there is an urgent need to explore how QPE is conceptualized and implemented in specific cultural and educational settings, which is crucial to ensure that the QPE instrument is reliable and valid. In particular, the centralized nature of the Chinese education system [23], creates a unique context for the implementation of QPE, potentially influencing individuals perceive and apply QPE.

To address gaps in the existing literature, our objective was to develop a QPE measurement instrument and assess its validity and reliability within the Chinese context, focusing specifically on students’ experiences. This approach aims to provide a valuable tool for evaluating the quality of PE programs and promoting lifelong PA among adolescents. Our study fills a crucial gap by incorporating students’ perspectives, as existing research has largely neglected their views. Additionally, we applied both EFA and CFA to ensure the instrument’s reliability and construct validity, offering a comprehensive solution to the limitations in previous studies.

## Methods

Our study consisted of three phases: In Phase 1, we used grounded theory with interviews with 22 PE teachers and 20 students to establish the conceptual framework for QPE. Phase 2 involved the creation of an item pool based on the conceptual framework established in Phase 1, supported by a literature review, expert evaluation, and student interviews. In Phase 3, we assessed the reliability and validity of this instrument in a sample of secondary school students. This study was conducted from November 2022 to October 2023. Ethical approval was obtained from the University of Malaya Research Ethics Committee (UM.TNC2/UMREC_2042), and in China, approval was granted by Chongqing Normal University. Written informed consent was obtained from the school principals and PE teachers. Prior to the students’ participation, written informed consent was also obtained from their parents or legal guardians. Furthermore, the students provided assent and signed their own written informed consent forms. All procedures adhered to the principles outlined in the Declaration of Helsinki.

### Phase 1: Conceptual framework development

Phase 1 aimed to develop a conceptual framework for QPE in the Chinese context using a grounded theory approach. For the number of participants, Thomson [24] recommended that 20 interviews were sufficient for grounded theory. Theoretical saturation occurs when no new concepts or categories emerge from additional data [25]. In this study, we initially analysed 40 interview transcripts and reserved 2 transcripts to confirm theoretical saturation. After completing the initial coding process on the 40 transcripts, we coded these final 2 interviews. Since no new concepts or categories emerged, we concluded that theoretical saturation had been reached.

Inclusion criteria for PE teachers included (a) In-service PE teachers in secondary schools, (b) teachers representing all age groups and varying lengths of teaching experience, and (c) willingness to participate in this study with a sign-on consent form. Teachers who did not meet these criteria or declined to provide informed consent were excluded from the sample. A total of 22 PE teachers (16 males and 6 females) were included were selected. Their ages ranged from 25 to 57 years (M = 38.23, SD = 8.40), and their teaching experience ranged from 2 to 36 years (M = 16.23, SD = 8.83). The participants were from junior and senior secondary schools located in the eastern, western, central, northern, and southern regions of China. We employed a purposive sampling method to recruit PE teachers for the in-depth interviews.

Inclusion criteria for students included (a) currently enrolled secondary school students, (b) participation in the school PE program, (c) no diagnosed physical or mental health conditions, and (d) willingness and ability to participate with their parental consent. A total of 20 students (10 males and 10 females) were selected. Their ages ranged from 11 to 14 years (M = 14.25, SD = 1.92). We employed a random sampling method to recruit students for the focus group interviews.

A senior-level PE teacher and two experts in PE and education refined the interview questions to align with this research objectives. The study included five key open-ended questions: (a) Could you describe your personal experiences and observations while teaching/learning PE classes? (b) In your opinion, what aspects contribute to improving the quality of PE? What areas do you think need further improvement? (c) What do you consider to be the primary challenges in implementing PE? (d) How do these challenges impact the effectiveness of PE teaching/learning? and (e) Based on your experience, what key dimensions or elements should QPE include? Due to geographical constraints, interviewee preferences, and the COVID-19 lockdown policy in China, all in-depth interviews were conducted online. Each interview lasted 40–50 min. After obtaining participants’ consent and permission to record, the process was audio-recorded and transcribed verbatim.

To ensure the accuracy of the transcriptions, this study employed a rigorous transcription process [26]. The initial transcription was completed using iFLYTEK professional transcription software, but all transcribed texts were manually proofread to ensure consistency with the original recordings. We repeatedly listened to the recordings during transcription and checked for omissions or errors. Additionally, all transcriptions were checked against the original recordings by two independent researchers, who compared any discrepancies and resolved them by re-listening to the audio. We confirmed the participants’ understanding of any unclear parts to ensure accuracy. Finally, a total of 42 transcripts were generated from the interviews, comprising 99,954 words.

The data were then analysed using grounded theory’s three-level coding approach using NVivo 12 software, which included open, axial, and selective coding. Specifically, during open coding, we (re)reviewed all interview and discussion transcripts line-by-line and the literature to identify recurring patterns and concepts [27]. We conducted a three-step open coding process: labelling, conceptualization, and categorization [28]. During the axial coding, the initial concepts were condensed and merged into more refined categories. This process involved examining and grouping conceptually similar codes, thereby distilling the data into higher-level themes [28]. More abstract higher-level categories were established using the constant comparative method. Specifically, the first and second authors held regular collaborative discussions during the axial coding phase. In these sessions, we compared codes and emerging categories, examined discrepancies, and made continuous theoretical and practical comparisons among categories. In the final step of selective coding, core categories were chosen as the main frameworks to structure other categories and phrases [28].

Moreover, member verification was conducted to enhance the reliability and credibility of the findings. Member checking was conducted by providing all participants with a summary of their analysis, along with a summary of the levels and categories contributing to QPE, based on the collective experiences of the participants. Subsequently, participants were asked to evaluate how well these descriptions matched their reported experiences. All 20 students agreed that the summaries were accurate. Of the 22 PE teachers, 17 agreed that the summaries were accurate. The feedback of the five teachers who disagreed was reviewed and discussed with them to ensure that necessary revisions were made and that the final descriptions were accurate.

### Phase 2: Item pool development

Phase 2 aimed to develop an item pool. First, we generated an initial item pool by reviewing relevant literature. Second, we refined the item pool through expert evaluations and student interviews.

The literature review was conducted to identify key concepts and dimensions of QPE, serving as a foundation for developing an item pool. Searches were performed across multiple databases, including China National Knowledge Infrastructure, Wanfang, Web of Science, Scopus, Google Scholar, and ProQuest, using keywords such as ‘quality physical education’, ‘quality of physical education’, and ‘high-quality physical education’, with year range from 2013 to 2024. After eliminating duplicated articles, 14 articles were left, the remaining 11 English-language academic articles [8,12,29–37] and three Chinese-language academic articles related to QPE [38–40]. Moreover, we relied on official documents when developing the item pool. They provide standards that help us select appropriate items. These documents include the *International Charter of Physical Education, Physical Activity and Sport* [41], the *Quality Physical Education: Guidelines for Policy-Makers* [7], the *Senior Secondary School Physical Education and Health Curriculum Standards* [42], the *Physical Education and Health Programme Standards for Compulsory Education* [43], the *Opinions on Comprehensively Strengthening and Improving School and University Physical Education Work in the New Era* [44], the *Guidelines for the Reform of Physical Education and Health Teaching (Trial Version)* [45], the *Notice on Enhancing the Quality of After-School Physical Education and Sports Training Services to Promote the Healthy Development of Primary and Secondary School Students* [46], the *Ensure the Implementation of the Regulation for One Hour of Daily Sport Activities in Primary and Secondary Schools* [47], and the *Opinions on Improving the Co-education Mechanism among Schools, Families and Society* [48].

We refined the item pool through a two-step process. First, six experts meeting the criteria of specialized knowledge in PE or physical literacy for adolescents, with at least five years of experience and either a master’s or PhD degree, were invited to provide feedback on the initial item pool. An email summarizing the study’s purpose, scope, and significance was sent, along with a consultation form containing demographic questions, a clarity rating form, and a QPE item relevance evaluation scale. Expert evaluations were conducted in two rounds: in the first round, three experts rated the clarity of each item on a 5-point Likert scale (1 = not at all clear, 5 = very clear). In the second round, based on the first round’s results, the remaining three experts rated the relevance of each item on a 4-point Likert scale (1 = not relevant, 4 = highly relevant). Second, two junior and one senior secondary school student were interviewed to assess their interpretation of the items. Participants raised concerns and suggested improvements for clarity, leading to further revisions of the subscales. This step-by-step process helped enhance this instrument’s content validity by evaluating the clarity and relevance of each item, aligning them with the constructs of the instrument that we aim to measure [49].

### Phase 3: Validity and reliability evaluation

Phase 3 aimed to assess the validity and reliability of items developed in Phase 2. A cross-sectional study was conducted to evaluate this instrument further. Principals of secondary schools were approached to gain permission for students to participate in a study. Participants were randomly sampled from the student population. We first created a list of all enrolled students. We then used a random number generator to select individuals from this list. Each selected individual received an invitation to participate in the study. The students were told that the study’s objective was to assess their experiences in PE, emphasising that the survey did not seek ‘right’ or ‘wrong’ answers. The students were informed about the survey’s voluntary nature and their freedom to withdraw at any time. Printed questionnaires were distributed and completed to students in a quiet classroom supervised by a researcher in the absence of their teachers to ensure an environment conducive to honest responses. Furthermore, the survey’s anonymity was highlighted, assuring students that all provided information would remain confidential. It was also explicitly stated that their responses would not be accessible to their teachers, reinforcing the privacy and confidentiality of their participation. Inclusion criteria for the student group were: (a) currently enrolled secondary school students, (b) participation in the PE program, (c) no diagnosed physical or mental health conditions, and (d) willingness and ability to participate with parental consent.

### Data analysis

Data analysis was conducted using IBM SPSS Statistics (Version 27) and IBM SPSS AMOS (Version 26). A bootstrap resampling procedure with 5,000 samples was implemented using AMOS. Bootstrapping is a robust technique that does not rely on the assumption of multivariate normality and can provide more accurate standard errors and confidence intervals under such conditions [50]. In the context of our study, bootstrapping contributed to the validity of the findings by allowing us to evaluate the precision of our parameter estimates and test the stability of the factor structure. This procedure also helps minimize the potential bias in the model’s estimates, strengthening the internal validity of the study’s conclusions [51].

We decided to conduct EFA separately for each subscale. This approach is justified by the robust guidance derived from Phase 1 of the grounded theory, which identified the conceptual framework of QPE for instrument development. This alignment facilitates the refinement of scales based on theoretical foundations and empirical evidence [52,53]. Moreover, previous studies have shown that key information may be overlooked if EFA is not conducted with care, potentially leading to a distorted factor structure [54,55]. This is a common pitfall of EFA since it is primarily data-driven and may capitalize on random variance within a given sample [56]. Moreover, separate EFAs are well-supported in studies [57–62]. We used principal component analysis (PCA) to explore the factor structure. PCA is particularly suited for data reduction and maximizing the explained variance of each component [63]. This data-driven approach uncovered the unique structural characteristics of QPE within its cultural context and avoided issues like factor indeterminacy [64]. As a result, PCA strengthened the instrument’s psychometric properties and improved its validity and reliability. The suitability of the data for factor analysis was assessed using the Kaiser-Meyer-Olkin (KMO) measure of sampling adequacy and Bartlett’s test of sphericity. KMO values above.60, and preferably.80, were considered acceptable, with Bartlett’s test requiring *p* < .05 [65]. This study identified factors having eigenvalues exceeding 1.0. Using Meyers et al. [66] as a guide, we opted for Promax rotation due to potential factor correlations, considering the potential correlations between factors. Items with loadings below.40 or significant cross-loadings (.32) were removed to ensure logical factor consistency [67]. Furthermore, according to the guidelines proposed by DeVellis [15] and Hair et al. [63], items that lack theoretical consistency with the respective factor and a factor with fewer than two items were excluded.

CFA was conducted to validate the instrument structure further, using fit indices such as Chi-square, comparative fit index (CFI), Tucker-Lewis index (TLI), standardised root mean square residual (SRMR), and root mean square error of approximation (RMSEA). A CFI and TLI > .90 and SRMR and RMSEA < .08 indicated an acceptable model fit [63]. Hair et al. [63] consider that items exhibiting standardised loadings below.50 in CFA are typically advised for removal. However, some social science research suggests that a standardized loading value reduced to.40 is considered acceptable [68,69]. Considering that this study involves the initial development of a measurement instrument, we have adopted the criterion of.40. Based on Brown’s [70] guidelines, correlations were added to the items’ residuals to enhance the model’s fit, starting with the highest modification index (MI) values and progressively addressing lower MI values until an acceptable model fit was achieved. These correlations were added within the same factor. Convergent validity was also assessed. Convergent validity was supported by factor loadings > .40, average variance extracted (AVE) >.40, and composite reliability (CR) >.60 [63,71].

We employed the CR and test-retest reliability to assess this instrument’s reliability. The CR was recommended by Fornell and Larcker [71], and the CR is considered a superior alternative to the traditionally used Cronbach’s alpha [72]. According to Hair et al. [63], an acceptable threshold for reliability is.70. We assessed the test-retest reliability of the instrument using the intraclass correlation coefficient (ICC) with a 2-way random-effects model. We calculated a 95% confidence interval (CI) to provide a more precise reliability estimate. ICCs range from 0 to 1, with values above.70 generally considered to indicate good reliability [73].

## Results

**Fig 1:**
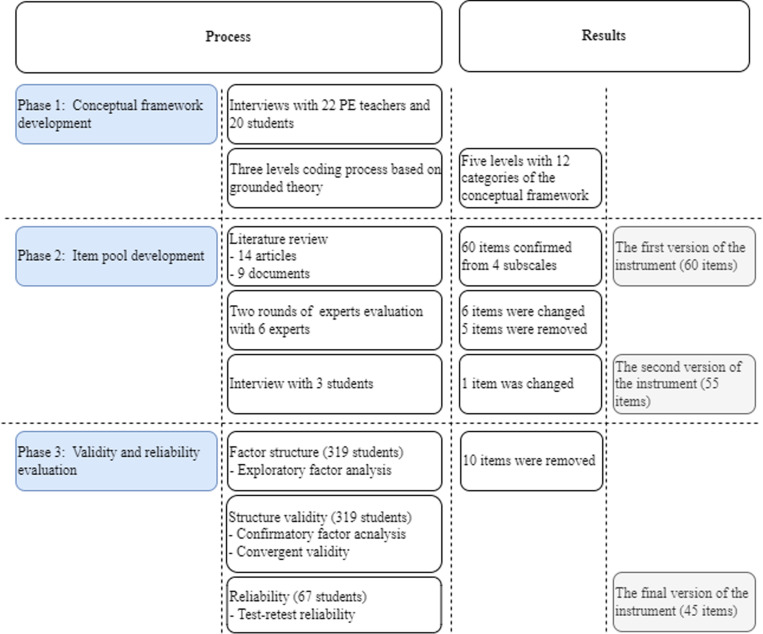
The process and results of each phase.

In Phase 1, in-depth and focus group interviews with 22 PE teachers and 20 students informed a grounded theory coding process, yielding a conceptual model with five levels and 12 categories. In Phase 2, a literature review provided the basis for creating a pool of 60 items aligned with four subscales. Two rounds of expert evaluation and an interview with three students led to item modifications and removals, reducing the instrument to 55 items and refining one additional item. In Phase 3, data from 319 participants underwent EFA, which removed 10 items and resulted in a final 45-item instrument. Based on CFA, convergent validity with a sample of 319 students, and test-retest reliability with 67 students, the findings supported the instrument’s psychometric properties. Fig 1 demonstrates the results of the three phases.

### Phase 1: Conceptual framework development

**Fig 2:**
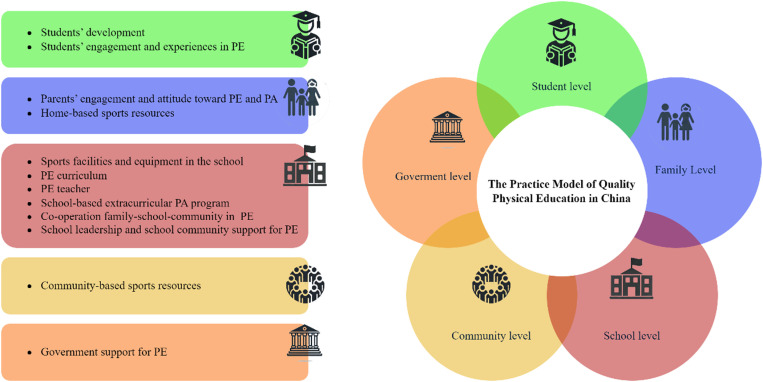
The practice model of quality physical education in China.

The initial phase of analysing interview data, open coding, involves conceptualizing, refining, and categorizing raw empirical information, particularly focusing on participants’ perspectives on QPE. This process begins with labelling relevant statements, generating 216 labels highlighting aspects like government policy, parents’ roles, and the quality of the PE curriculum (S1 Table). These labelled statements are then refined through conceptualization, resulting in 51 concepts (S2 Table). Categorization follows, identifying 12 categories through ongoing analysis and inductive reasoning (S3 Table). The subsequent axial coding phase connects and further develops these categories, examining causal relationships, interaction strategies, and key phenomena. Five levels were identified: student, family, school, community, and government levels. Each level entails categories, such as student engagement, parental attitudes, and government support (S4 Table). The final stage, selective coding, identifies a core concept that integrates all categories, named ‘The Practice Model of Quality Physical Education in China’. The three-level coding process, which includes labelling, conceptualization, categorization, and forming main and core categories, ultimately resulted in the development of a QPE practice model tailored to the Chinese context (Fig 2).

### Phase 2: Item pool development

Adolescence is characterized as a period of significant biological and psychosocial change [74]. They may possess relatively insufficient knowledge and experiential background with respect to government operations, public policy, and social structures. Consequently, item design focusing on the government level will not be included in the items pool development. This approach led to the development of the first version of the instrument, structured across four subscales: student, family, school, and community.

The student subscale included students’ development (9 items) and engagement and experience in PE (5 items). The family subscale included parents’ engagement and attitudes toward PE and PA (7 items) and home-based sports resources (5 items). The school subscale included sports facilities and equipment in the school (3 items), PE curriculum (10 items), PE teacher (5 items), school-based extracurricular PA programs (4 items), co-operation family-school-community in PE (3 items), and school leadership and school community support in PE (3 items). The community subscale included community-based sports resources (6 items). A detailed description of items is provided in S5 Table.

Based on the first round of expert assessment (S6 Table), items scoring below an average of 3.5 were identified as lacking clarity, leading to the revision of five items according to expert feedback. SCL 7 was revised to ‘The PE class adopts a multifaceted assessment approach to measure my performance’, while SCL 8 was changed to ‘There are equal opportunities for all students to practice in PE’. SCL 13 was modified to ‘Structured PA during PE classes encourages my active participation, allowing me to engage in exercises that lead to sweating’. SCL 16 was updated to ‘The PE teacher accurately demonstrates motor and sports techniques and shows strong professional competence’, and SCL 26 was revised to ‘The school promotes a family-school-community collaborative PE program and organizes sports events’. Additionally, one expert noted that at the school subscale, SCL 28, originally described as ‘Schools often use community sports grounds and facilities for PE programs’, did not fully align with the context in China, where community collaboration in PE is minimal. The item was thus revised to ‘The school has implemented PE homework supervised by parents at home’ to better reflect the situation in Chinese schools. The second round of expert evaluations evaluated all items. Five items, STL 12, FL 11, FL 12, SCL 14, and CL 3, exhibited an I-CVI of.66 or 0 (S7 Table) and were subsequently removed.

Three secondary school students (two males and one female) were invited to participate in interviews to verify the validity of the items and the clarity of language. The mean age of participants was 14. Items were modified based on participants’ feedback regarding presentation, wording, option setting, and question annotation of the subscale items. One item at the school subscale was revised after the secondary student interviews, SCL 8: ‘There are equal opportunities for practice in PE’, which was amended to ‘In PE, I have the equal opportunities to practice as other students’.

After the development process was concluded, the second version of the instrument consisted of 55 items: the student subscale (13 items), the family subscale (10 items), the school subscale (27 items), and the community subscale (5 items). The items in the study were rated using a 7-point Likert scale ranging from 1 (strongly disagree) to 7 (strongly agree). We employed a 7-point Likert scale because prior research indicates that it more effectively captures participants’ psychological characteristics compared to a 5-point Likert scale [75].

### Phase 3: Validity and reliability evaluation

Danielsoper, an online sample size calculation platform [76], was utilized to determine the required sample size. The expected effect size was set at.30, the desired statistical power level at.80, and the significance level at.05. The analysis was performed based on selecting these 27 observed and six latent variables from the largest scale. With these parameters, the minimum sample size required for CFA is 200.

Seven hundred-five secondary school students from Chongqing, a metropolitan area in southwestern China, were recruited sequentially across three sample groups. Sample 1, which consisted of 319 participants (59.3% males, 40.7% females) aged 12–18 years (mean: 14.78, SD: ± 1.60), was used for EFA. Sample 2, which comprised 319 students (48.3% male, 51.7% female) aged 12–19 years (mean: 14.93, SD: ± 1.64), was used for CFA. Sample 3, which comprised 67 students (42% male, 58% female) aged 12–19 years (mean: 13.91, SD: ± 1.68), was used for test-retest reliability.

#### Exploratory factor analysis.

The student subscale initially contained 13 items. After one round of EFA, no item was removed. An adequate KMO value and signiﬁcant Bartlett’s test of sphericity for the subscale (.909, χ2 = 2525.561, *p* < .001) and a two-factor structure solution with eigenvalues greater than 1.0 were extracted with 64.17% of the total variance explained. The extracted factors were the following: students’ development, and students’ engagement and experience in PE. The family subscale initially contained 10 items. After three rounds of EFA, three items were removed: two items (FL 7 and FL 10) were excluded because the factor contained fewer than two items, and one item (FL 6) was removed due to a lack of theoretical consistency with the factor. The family subscale showed satisfactory KMO and Bartlett’s test of sphericity values (.841, χ2 = 1265.231, *p* < .001). Two factors were extracted with 73.39% of the total variance. The two identiﬁed factors were parents’ involvement in PA and home-based sports resources, and parents’ attitude toward PE. The school subscale initially contained 27 items. After five rounds of EFA, seven items were removed: two items (SCL 11 and SCL 13) were excluded due to cross-loading, four items (SCL 8, SCL 23, SCL 24 and SCL 25) because they loaded below.4, and one item (SCL 21) due to a lack of theoretical consistency with the factor. The satisfactory KMO value of.891 and Bartlett’s sphericity value of χ2 = 3776.946, *p *< .001 for the school subscale, indicated the factorability of the items. Five factors were extracted with 68.67% of the total variance. Five factors include concepts of sports facilities and equipment in the school, PE curriculum, PE teacher, school-based extracurricular PA program, co-operation family-school-community in PE. The community subscale initially contained five items. After one round of EFA, no item was removed. An adequate KMO value and signiﬁcant Bartlett’s test of sphericity for the subscale (.880, χ2 = 1435.467, *p* < .001) and a one-factor structure solution with eigenvalues greater than 1.0 were extracted with 80.54% of the total variance explained. The extracted factor was the following: community-based sports resources. All factor loadings were signiﬁcant with values greater than.40, indicating an adequate proportion of common variance among the items in each subscale. The factor loading values and items loaded into each identiﬁed EFA factor are shown in Table 1.

**Table 1:** Item loadings for four subscales of the instrument.

Factors	Items	Factor Loading	KMO Test	*p* value	Sums of Squared Loadings
**Student subscale**			0.909	< 0.001	64.17%
Students’ development	STL 9	0.867			
	STL 1	0.853			
	STL 2	0.838			
	STL 3	0.821			
	STL 6	0.820			
	STL 8	0.819			
	STL 5	0.740			
	STL 4	0.676			
	STL 7	0.651			
Students’ engagement and experience in PE	STL 14	0.863			
	STL 11	0.827			
	STL 10	0.822			
	STL 13	0.669			
**Family subscale**			0.841	< 0.001	73.39%
Parents’ involvement in PA and home-based sports resources	FL 4	0.923			
	FL 5	0.857			
	FL 9	0.798			
	FL 8	0.771			
Parents’ attitude toward PE	FL 3	0.909			
	FL 2	0.854			
	FL 1	0.823			
**School subscale**			0.891	< 0.001	68.67%
Sports facilities and equipment in the school	SCL 2	0.834			
	SCL 3	0.760			
	SCL 1	0.638			
PE curriculum	SCL 5	0.912			
	SCL 10	0.904			
	SCL 6	0.760			
	SCL 9	0.687			
	SCL 4	0.649			
	SCL 7	0.599			
	SCL 12	0.591			
PE teacher	SCL 18	0.912			
	SCL 15	0.889			
	SCL 16	0.876			
	SCL 17	0.862			
School-based extracurricular PA program	SCL 19	0.915			
	SCL 20	0.792			
	SCL 22	0.612			
Co-operation family-school-community in PE	SCL 28	0.972			
	SCL 27	0.901			
	SCL 26	0.846			
**Community subscale**			0.880	< 0.001	80.54%
Community-based sports resources	CL 2	0.923			
	CL 1	0.913			
	CL 5	0.897			
	CL 6	0.888			
	CL 4	0.866			

Note: STL = Student level, FL = Family level, SCL = School level, CL = Community level

#### Confirmatory factor analysis.

**Fig 3:**
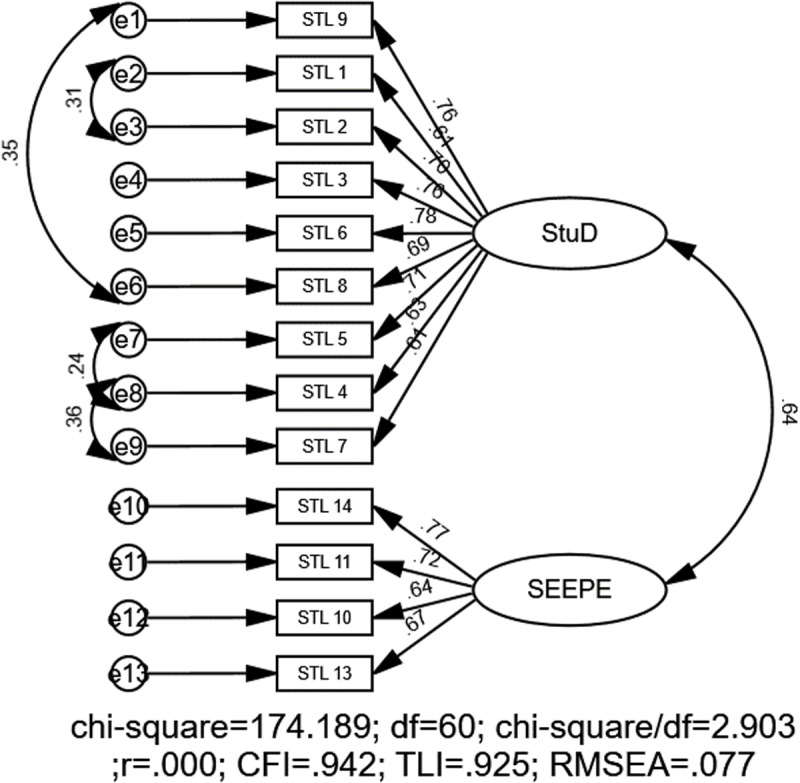
CFA model for the student subscale. Note: STL = Students level, SD = Students’ development, SEEPE = Students’ engagement and experience in PE.

For the student subscale, the initial RMSEA index indicated potential for model improvement, with χ2 = 293.45, *df* = 64, CFI = .884, TLI = .859, SRMR = .059, and RMSEA = .10 (90% CI:.09 to.11). To enhance the model’s fit, error covariances were added between items’ residuals, beginning with the pairs that had the highest MI values. The MIs from the CFA indicated significant potential error covariances between STL 4 and 7 (MI = 40.672), 8 and 9 (MI = 29.368), 1 and 2 (MI = 24.212), as well as 4 and 5 (MI = 16.607). This iterative process led to the final model, which demonstrated satisfactory goodness-of-fit indices: χ2 = 174.18, *df *= 60, CFI = .942, TLI = .925, SRMR = .049, and RMSEA = .077 (90% CI:.06 to.09) (Fig 3).

**Fig 4:**
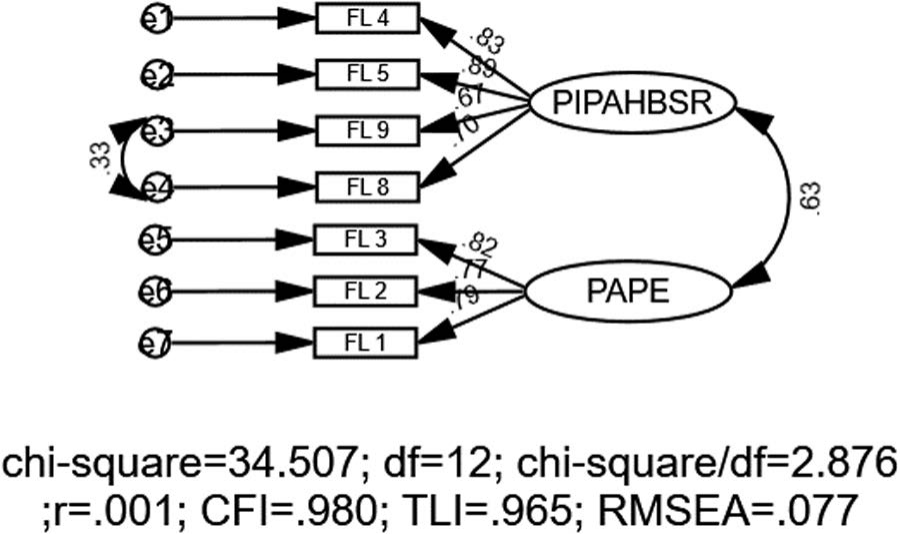
CFA model for the family subscale. Note: FL = Family level, PIPAHBSR = Parents’ involvement in PA and home-based sports resources, PAPE = Parents’ attitude toward PE.

For the family subscale, the initial model fit indices showed room for refinement, with χ2 = 59.99, *df* = 13, CFI = .958, TLI = .933, SRMR = .043, and RMSEA = .107 (90% CI:.08 to.13). To enhance the model’s fit, error covariances were added between items’ residuals, beginning with the pairs that had the highest MI values. The MIs from the CFA indicated significant potential error covariances between FL 8 and 9 (MI = 20.352). This iterative process led to the final model, which demonstrated satisfactory goodness-of-fit indices: χ2 = 43.50, *df *= 12, CFI = .980, TLI = .965, SRMR = .047, and RMSEA = .077 (90% CI:.04 to.10) (Fig 4).

**Fig 5:**
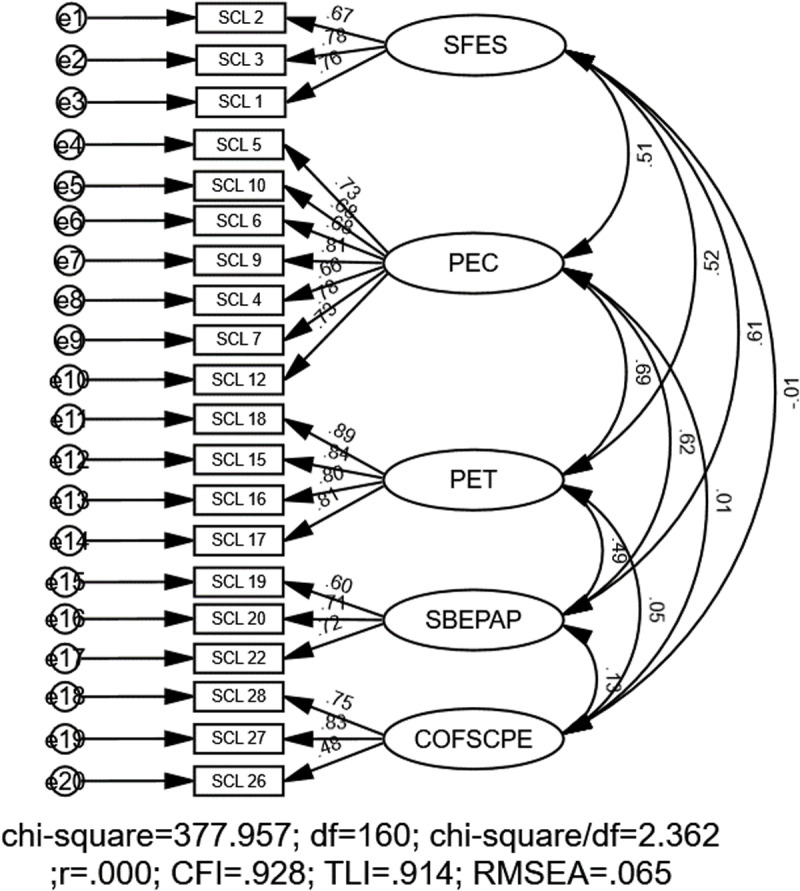
CFA model for the school subscale. Note: SCL = School level, SFES = Sports facilities and equipment in the school, PEC = Physical education curriculum, PET = Physical education teacher, SBEPAP = School-based extracurricular physical activity program, COFSCPE = Co-operation family-school-community in PE.

For the school subscale, the initial fit indices suggested good model fit, with χ2 = 377.95, *df* = 160, CFI = .928, TLI = .914, SRMR = .050, and RMSEA = .065 (90% CI:.05 to.07) (Fig 5).

**Fig 6:**
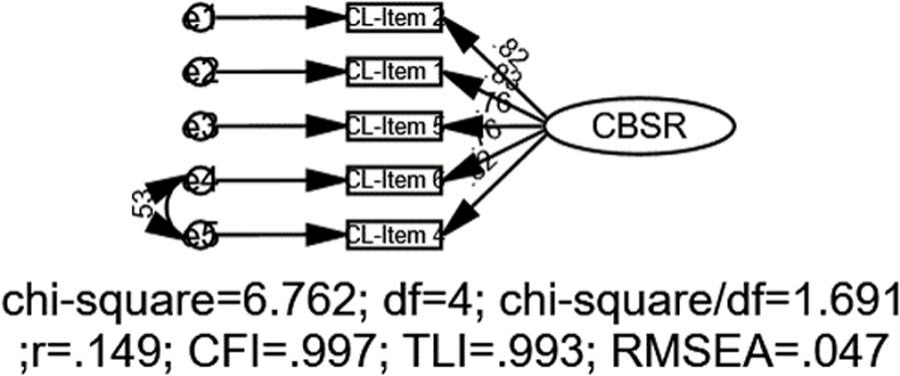
CFA model for the community subscale. Note: CL = Community level, CBSR = Community-based sports resources.

For the community subscale, the initial model showed suboptimal fit indices, with χ2 = 60.37, *df* = 5, CFI = .946, TLI = .891, SRMR = .044, and RMSEA = .187 (90% CI:.14 to.23). To enhance the model’s fit, error covariances were added between items’ residuals, beginning with the pairs that had the highest MI values. The MIs from the CFA indicated significant potential error covariances between CL 4 and 6 (MI = 20.066). This iterative process led to the final model, which demonstrated satisfactory goodness-of-fit indices: χ2 = 6.76, *df *= 2, CFI = .997, TLI = .993, SRMR = .013, and RMSEA = .047 (90% CI:.000 to.10) (Fig 6).

#### Convergent validity.

Table 2 presents the convergent validity of the instrument. The AVE values across the 10 factors ranged from.462 to.698, while the CR values ranged from.719 to.902. These results demonstrate that the instrument exhibits acceptable convergent validity. Furthermore, the CR values exceed.70, indicating the instrument has good reliability.

**Table 2:** Convergent validity of the instrument.

Factors	CR	AVE
**Student subscale**		
Students’ development	0.895	0.488
Students’ engagement and experience in PE	0.792	0.489
**Family subscale**		
Parents’ involvement in PA and home-based sports resources	0.858	0.606
Parents’ attitude toward PE	0.835	0.628
**School subscale**		
Sports facilities and equipment in the school	0.781	0.545
PE curriculum	0.881	0.516
PE teacher	0.902	0.698
School-based extracurricular PA program	0.719	0.462
Co-operation family-school-community in PE	0.736	0.494
**Community subscale**		
Community-based sports resources	0.896	0.633

After the EFA and CFA, the final version of the instrument consisted of 45 items in 10 factors with four subscales: (a) The student subscale consisted of students’ development (9 items), and students’ engagement and experience in PE (4 items). (b) The family subscale consisted of parents’ involvement in PA and home-based sports resources (4 items), and parents’ attitude toward PE (3 items). (c) The school subscale consisted of sports facilities and equipment in school (3 items), PE teacher (4 items), PE curriculum (7 items), school-based extracurricular PA program (3 items), and co-operation family-school-community in PE (3 items). (d) The community subscale consisted of community-based sports resources (5 items).

#### Test-retest reliability.

This study used an independent sample of 67 secondary students comprising 33 males and 34 females. Participants ranged from 12 to 18 years, with a mean age of 13.91 ± 1.68. These students completed the instrument on two separate occasions spaced three weeks apart. Table 3 demonstrates good test-retest reliability across all 10 factors, with ICCs consistently exceeding.70 (range:.743-.905) and 95% confidence intervals not encompassing zero. These results indicate strong temporal stability and measurement consistency.

**Table 3:** Test-retest reliability.

Factors	Test 1: Mean ± SD	Test 2: Mean ± SD	ICC (95% CI)
Students’ development	54.00 ± 8.90	56.04 ± 8.36	0.864 (0.788-0.918)
Students’ engagement and experience in PE	23.41 ± 4.80	25.00 ± 3.78	0.905 (0.850-0.941)
Parents’ involvement in PA and home-based sports resources	19.25 ± 6.31	21.43 ± 6.15	0.787 (0.674–0.864)
Parents’ attitude toward PE	17.16 ± 3.83	18.04 ± 3.33	0.875 (0.804–0.922)
Sports facilities and equipment in the school	15.91 ± 3.55	16.71 ± 3.94	0.829 (0.735–0.892)
PE curriculum	39.88 ± 6.99	41.52 ± 7.82	0.796 (0.688–0.870)
PE teacher	24.58 ± 3.63	25.31 ± 3.49	0.748 (0.619–0.838)
School-based extracurricular PA programs	18.20 ± 3.01	18.40 ± 3.41	0.856 (0.775–0.909)
Co-operation family-school-community in PE	12.64 ± 5.76	14.43 ± 6.45	0.743 (0.612–0.834)
Community-based sports resources	22.65 ± 8.13	26.97 ± 7.31	0.744 (0.614–0.835)

## Discussion

This study aimed to develop and validate an instrument for assessing the implementation of QPE using scientific procedures. The final instrument has 45 items, divided into four subscales across 10 factors: students’ development, students’ engagement and experience in PE, parents’ involvement in PA and home-based sports resources, parents’ attitude toward PE, sports facilities and equipment in the school, PE curriculum, PE teacher, school-based extracurricular PA programs, co-operation family-school-community in PE, and community-based sports resources. The psychometric assessment provided sound evidence for its validity and reliability in evaluating QPE perception among secondary school students. Our results showed acceptable content, construct validities and reliability.

### Measurement characteristics

In this study, a three-phase process was used to develop a QPE measurement instrument for China. In Phase 1, a conceptual framework of QPE in China was developed through interviews and grounded theory coding, resulting in five levels and 12 categories. In Phase 2, a pool of 60 items was created by reviewing 14 articles and nine documents. Expert evaluations and student interviews were conducted to refine the items, leading to a revised instrument with 55 items across four subscales (student, family, school, community). In Phase 3, EFAs were performed for each subscale using PCA to explore the factor structure. PCA helped reduce data and maximize variance explained, ensuring the model’s validity and reliability. Items were excluded due to low factor loadings, theoretical inconsistencies, or insufficient item representation per factor (i.e., fewer than two items), resulting in a refined instrument consisting of 45 items. These steps ensure strong associations between the measured items and their underlying factors. We added error covariances between items’ residuals to enhance the model’s fit, starting with the pairs with the highest MI values. According to Kline [77], potential error covariances can arise when items are worded similarly or involve overlapping content that could influence responses independently of the latent construct. In this study, all MI modification paths were added within the same factor because the items in each pair measure the same construct. Correlating items within the same factor is acceptable, as they are designed to assess the same underlying latent construct. Therefore, these adjustments are justified [77]. The instrument demonstrated strong psychometric properties, including convergent validity (AVE > .40, CR > .60), internal consistency (CR > .70), and test-retest reliability, confirming its suitability for measuring QPE across repeated assessments.

### A comparison of existing QPE instruments

Our findings aligns with Ho et al.’s [12] and He et al.’s [13] questionnaires on several key dimensions, including teaching quality and sports resources. Both our study and Ho et al. [12] emphasize the importance of social and cultural factors. While Ho et al. [12] focused on social norms, our study expands this by addressing parents’ involvement, family-school-community co-operation, and community-based sports resources.

However, our study differs from Ho et al. [12] and He et al, [13] in the following ways: First, we developed an instrument that targets the school environment and external factors influencing students’ engagement in PE and PA. Our instrument encompasses a broader ecological framework, including students, family, school, and community. This holistic approach aligns with studies that advocate for interventions in PE and PA to extend beyond the school setting [7,78,79]. Research indicates that it is crucial to consider the social and ecological environments in which students live.

Second, compared to Ho et al.’s [12] study, our research aligns more closely with the Chinese context because it directly incorporates the perspectives of Chinese PE teachers and students through in-depth and focus group interviews. These methods enabled us to develop a QPE conceptual framework that resonates with local policy guidelines, sociocultural values, and pedagogical practices. By tailoring the survey items to the specific experiences of Chinese stakeholders, the resulting measurement instrument reflects the unique institutional, curricular, and cultural realities of PE in China, thereby ensuring greater contextual relevance and applicability.

Third, the research of He et al. [13] developed items based solely on one existing Chinese policy. While policy analysis is important, relying exclusively on policies may lead to a limited perspective. This approach might overlook the practical experiences and insights of educators and students in the field. By contrast, our study adopted a more holistic strategy. We constructed a QPE conceptual framework grounded in interviews with teachers and students, ensuring that practical insights underpinned our theoretical model. Subsequently, we consulted pertinent literature and policy documents to refine our item pool, thus capturing both empirical and policy-oriented dimensions of QPE in China. In addition, He et al. [13] performed EFA without subsequent CFA, we conducted EFA and CFA separately for each subscale (student, family, school, and community). By adopting this method, we offer a more robust and contextually relevant instrument for assessing the different dimensions of QPE. This method contributes to the field by providing a theoretically sound and empirically validated instrument.

Finally, while previous studies gathered insights from professionals and educators [12,13], our study centres on students as the main respondents. This approach recognizes students as critical stakeholders whose experiences and perceptions are vital for assessing the QPE implementation.

### Factors of influence on QPE in China

In this study’s context, the developed instrument with its four subscale, 10 factors, and 45 items can be further interpreted through the lens of McLeroy’s ecological model [80], which emphasizes the dynamic interplay between individual and environmental factors at multiple levels. This model can provide a comprehensive framework for understanding the influences on students’ PE experiences and PA behaviours.

For the student subscale, the instrument assesses students’ development, engagement, and experiences in PE, which aligns with McLeroy’s (1988) focus on intrapersonal factors. This level reflects individual characteristics, including knowledge, attitudes, behaviours, self-concept, and skills. It also encompasses the individual’s developmental history. Research has shown that fostering students’ development in PE is critical to promoting lifelong PA. For instance, building a strong foundation in motor skills has been associated with increased participation in PA throughout life [78]. Additionally, students’ experiences and engagement in PE are pivotal in shaping their attitudes toward PA [19].

For the family subscale, the instrument incorporates items on parents’ involvement in PA and home-based sports resources, as well as parents’ attitudes toward PE, which aligns with McLeroy’s [80] focus on interpersonal processes and primary groups. This level reflects the formal and informal social networks and social support systems. Matos et al. [81] found that parents’ PA serves as a key model for their children’s PA behaviour. Additionally, promoting PA within the family context (instrumental or direct support) is important to enhance children’s overall PA levels [81].

For the school subscale, the instrument assesses key factors such as sports facilities and equipment, PE teachers, PE curriculum, school-based extracurricular PA programs, and co-operation family-school-community in PE. These factors correspond to the institutional elements described by McLeroy et al. [80], encompassing influences related to the school, workplace, or university environment, including influences from teachers, school administrators, and other institutional stakeholders. Numerous studies have highlighted the significance of sports facilities and equipment, PE teachers, and the PE curriculum as essential components of PE [12,82,83]. These factors affect students’ experiences in PE and influence broader outcomes such as motivation, self-efficacy, and academic performance [84–86]. The school-based extracurricular PA programs provide additional opportunities for students to engage in PA beyond regular PE classes [87], contributing to improved fitness and overall well-being. In China, school-based PA programs are essential as they are mandated by government policies [88], which require schools to offer extracurricular PA opportunities. The existing literature suggests that effective collaboration among families, schools, and communities promotes students’ study outcomes [89]. This finding aligns with the factor of co-operation between family-school-communities in PE, which was identified in the development of the present instrument, highlighting the importance of such collaboration in QPE. In addition, the school subscale encompasses the greatest number of QPE factors compared to the other three subscales (students, family, and community), which aligns with China’s three-level education system [23]. The central government formulates educational policies, local governments regulate their implementation in accordance with these directives, and schools serve as the primary institutions responsible for practical enactment. Therefore, the success of PE largely depends on schools’ capacity to translate these policies into practical actions, supported by the resources and infrastructure provided by both local and central government authorities.

For the community subscale, the instrument incorporates items on community-based sports resources, which aligns with McLeroy’s [80] focus on the community factor. This level focuses on modifying the community environment or services and organisation relationships. Research has shown that when communities invest in sports infrastructure and provide organized programs, individuals are more likely to engage in PA [90]. For example, communities with accessible parks, sports clubs, and recreational spaces have seen higher participation rates in PA [91]. This highlights the significance of ensuring that community-based sports resources are available and well-maintained, particularly in underserved areas [92]

### Significance of this study

This instrument offers several advantages. First, the Chinese government has introduced multiple policies to enhance PA and promote inclusive, high-quality PE. However, many objectives and strategies remain vague, making it difficult to translate broad, macro-level goals into practical school-based actions. Our QPE instrument bridges this gap by offering a clear framework of essential factors and levels. Schools can use it to systematically assess their PE programs and pinpoint areas for improvement. Second, the instrument is both reliable and valid for evaluating secondary students’ perceptions of QPE. It serves as a crucial instrument for assessing QPE program impact and effectiveness. By highlighting strengths and areas for improvement, it also guides resource allocation, enabling policymakers to identify schools or regions needing additional support. Educators and administrators can use these insights to refine pedagogical strategies, optimizing PE and PA programs. Additionally, the instrument also allows for monitoring QPE progress, aligning the curriculum with national goals. While developed for China, its universal elements can be adapted through translation or modified items or factors, enabling cross-country comparisons. This adaptability highlights the specific needs and contexts of diverse educational systems.

### Limitations and future research

Several limitations in this study warrant discussion. First, our interviews were conducted with PE teachers at the secondary school level (grades 7–12). As a result, the findings may not be generalizable to primary schools (grades 1–6) or universities. Future research should explore the experiences and perceptions of PE teachers across different educational stages, including primary schools and universities, as well as other PE professionals. Expanding the sample size to encompass various regions could also enhance the generalizability of the findings. Second, the participant selection poses a potential limitation because it included only secondary school students in China. Future research should include primary school and university students to assess the stability of the developed measurement instrument across different age groups. Third, because this instrument was originally developed in Chinese, its applicability and validity in different cultural contexts cannot be ensured. Further testing of this instrument to consider expanding the sample size across various regions and age demographics. Finally, the cross-sectional design restricts the capacity to establish causal relationships or observe changes over time. Future research should consider utilizing longitudinal or experimental designs to enhance reliability and validity assessment.

## Conclusion

This study develops and validates a self-reported instrument comprising 48 items distributed across 10 dimensions and four subscales to assess secondary students’ perceptions of QPE. The findings demonstrate that the instrument is reliable and valid for evaluating the implementation of QPE in secondary schools. Researchers, schools, and policy-makers can widely use the instrument to monitor and assess QPE programs. However, the instrument’s applicability across diverse educational contexts requires further validation, and the cross-sectional design limits its ability to track changes over time. Future research should examine the instrument’s cross-cultural applicability and explore its longitudinal use. This instrument represents a significant step forward in QPE assessment, offering valuable insights into secondary school and providing a foundation for future PE policy and practice improvements.

## Supporting information

S1 Table Labelling results. (DOCX)

S2 Table Conceptualisation results of 216 label statements. (DOCX)

S3 Table Open coding results. (DOCX)

S4 Table Results of the axis coding for 12 categories. (DOCX)

S5 Table Description of items. (DOCX)

S6 Table Assessment of the items’ mean values. (DOCX)

S7 Table Assessment of the items’ content validity index. (DOCX)

S8 Table Raw data. (XLSX)

## References

[1] World Health Organization. Global status report on physical activity 2022. 2022. Available from: https://www.who.int/teams/health-promotion/physical-activity/global-status-report-on-physical-activity-2022

[2] Huo M, Yang Z. Exercise, Depression, and Anxiety in Young People: A Cross-Sectional Survey. International Journal of Mental Health Promotion. 2023;25(4):551–62. doi: 10.32604/ijmhp.2023.023406

[3] Muntaner-Mas A, Morales JS, Martínez-de-Quel Ó, Lubans DR, García-Hermoso A. Acute effect of physical activity on academic outcomes in school-aged youth: A systematic review and multivariate meta-analysis. Scand J Med Sci Sports. 2024;34(1):e14479. doi: 10.1111/sms.14479 37632197 PMC10952189

[4] Li W, Xiang P. A Review on the Dose Response Effect of Regular Physical Activity on Cognitive Function Among Children and Adolescents. IJPAH. 2023. doi: 10.18122/ijpah.020203.boisestate

[5] Aubert S, Barnes JD, Demchenko I, Hawthorne M, Abdeta C, Abi Nader P, et al. Global Matrix 4.0 Physical Activity Report Card Grades for Children and Adolescents: Results and Analyses From 57 Countries. J Phys Act Health. 2022;19(11):700–28. doi: 10.1123/jpah.2022-0456 36280233

[6] National Association for Sport and Physical Education. NASPE resource brief: quality physical education. 2013 [cited 12 Aug 2023]. Available from: https://files.eric.ed.gov/fulltext/ED541490.pdf

[7] UNESCO. Quality physical education: Guidelines for policy-makers. Paris, France: UNESCO Publishing; 2015. Available from: https://unesdoc.unesco.org/ark:/48223/pf0000231101?posInSet=1&queryId=a0e81878-870f-46ad-89cd-200e39b99fef

[8] Williams J, Pill S. What does the term ‘quality physical education’ mean for health and physical education teachers in Australian Capital Territory schools? European Physical Education Review. 2018;25(4):1193–210. doi: 10.1177/1356336x18810714

[9] Le Masurier G, Corbin CB. Top 10 Reasons for Quality Physical Education. Journal of Physical Education, Recreation & Dance. 2006;77(6):44–53. doi: 10.1080/07303084.2006.10597894

[10] Dudley D, Mackenzie E, Van Bergen P, Cairney J, Barnett L. What Drives Quality Physical Education? A Systematic Review and Meta-Analysis of Learning and Development Effects From Physical Education-Based Interventions. Front Psychol. 2022;13:799330. doi: 10.3389/fpsyg.2022.799330 35846697 PMC9280720

[11] Mitchell SA, Walton-Fisette JL. The essentials of teaching physical education: curriculum, instruction, and assessment. Champaign: Human Kinetics. 2022.

[12] Ho WKY, Ahmed MdD, Kukurova K. Development and validation of an instrument to assess quality physical education. Cogent Education. 2021;8(1). doi: 10.1080/2331186x.2020.1864082

[13] He L, Ayub AFBM, Amri SB. Development and validation of a questionnaire to assess the implementation of physical education programs in Chinese junior high schools. BMC Public Health. 2024;24(1):2387. doi: 10.1186/s12889-024-19844-5 39223514 PMC11370090

[14] Ministry of Education, China. Student physical health monitoring and evaluation methods and other three documents [Annex: evaluation index system for physical education work in primary and secondary schools]. 2014 [cited 12 Oct 2024]. Available from: http://www.moe.gov.cn/srcsite/A17/s7059/201404/t20140428_168528.html

[15] DeVellis RF, Thorpe CT. Scale development: theory and applications. SAGE Publications; 2021.

[16] Amerstorfer CM, Freiin von Münster-Kistner C. Student Perceptions of Academic Engagement and Student-Teacher Relationships in Problem-Based Learning. Front Psychol. 2021;12:713057. doi: 10.3389/fpsyg.2021.713057 34777094 PMC8580851

[17] Cho HJ, Melloch MR, Levesque-Bristol C. Enhanced student perceptions of learning and performance using concept-point-recovery teaching sessions: a mixed-method approach. IJ STEM Ed. 2021;8(1). doi: 10.1186/s40594-021-00276-1

[18] Lu D. Students’ Perceptions of a Blended Learning Environment to Promote Critical Thinking. Front Psychol. 2021;12:696845. doi: 10.3389/fpsyg.2021.696845 34248799 PMC8267247

[19] Mercier K, Simonton K, Centeio E, Barcelona J, Garn A. Middle school students’ attitudes toward physical activity and physical education, intentions, and physical activity behavior. European Physical Education Review. 2022;29(1):40–54. doi: 10.1177/1356336x221106051

[20] Canada’s Physical and Health Education. Quality physical education. 2023 [cited 16 Aug 2023]. Available from: https://phecanada.ca/activate/qpe

[21] Department of Education, Australia. Quality physical education. 2021 [cited 11 Jan 2024]. Available from: https://www.education.vic.gov.au/school/teachers/teachingresources/discipline/physed/Pages/qualityphysed.aspx

[22] Office for Standards in Education, Children’s Services and Skills. A level playing field – all pupils entitled to high-quality PE. 2022 [cited 16 Aug 2023]. Available from: https://www.gov.uk/government/news/a-level-playing-field-all-pupils-entitled-to-high-quality-pe

[23] Meng X, Horrell A, McMillan P, Chai G. ‘Health First’ and curriculum reform in China: The experiences of physical education teachers in one city. European Physical Education Review. 2020;27(3):595–612. doi: 10.1177/1356336x20977886

[24] Thomson SB. Sample size and grounded theory. 5. 2010;1:45–52.

[25] Wutich A, Beresford M, Bernard HR. Sample Sizes for 10 Types of Qualitative Data Analysis: An Integrative Review, Empirical Guidance, and Next Steps. International Journal of Qualitative Methods. 2024;23. doi: 10.1177/16094069241296206

[26] Robinson O, Wilson A. Transcribing and coding. UBC Library; 31 Aug 2022 [cited 14 Feb 2023]. Available from: https://pressbooks.bccampus.ca/undergradresearch/chapter/transcribing-and-coding/.

[27] Strauss AL, Corbin JM. Basics of qualitative research: techniques and procedures for developing grounded theory. 2nd edition. Thousand Oaks: Sage Publications; 1998.

[28] Vollstedt M, Rezat S. An introduction to grounded theory with a special focus on axial coding and the coding paradigm. Compendium for early career researchers in mathematics education. 2019;13:81–100. doi: 10.1007/978-3-030-15636-7_4

[29] Ahmed MD, Heydari R, Kukurová K, Esfahani M, Ho WKY. A quantitative analysis measuring professionals’ perception about quality physical education (QPE). Cogent Education. 2023;10:2248887. doi: 10.1080/2331186x.2023.2248887

[30] Chen W, Mason S, Hypnar A, Hammond-Bennett A. Association of Quality Physical Education Teaching with Students’ Physical Fitness. J Sports Sci Med. 2016;15(2):335–43. 27274673 PMC4879449

[31] Del Val Martín P, Kukurová K, Ho W, Blázquez Sánchez D, Sebastiani Obrador EM. La percepción de la Educación Física de calidad (EFC) desde la óptica de los profesionales en Ecuador (The perceptual understanding of quality Physical Education (QPE) from professional in Ecuador). Retos. 2023;48:16–23. doi: 10.47197/retos.v48.96531

[32] González-Rivas RA, Núñez Enríquez O, Zueck-Enríquez MDC, Gastelum-Cuadras G, Ramírez-García AA, López-Alonzo SJ, et al. Analysis of the Factors That Influence a Quality Physical Education in Mexico: School Supervision’s Perspective. Int J Environ Res Public Health. 2022;19(5):2869. doi: 10.3390/ijerph19052869 35270561 PMC8910645

[33] Ho W, Ahmed M, Branislav A, Makszin I, Valeiro M, Kougioumtzis K. Development of an instrument to assess perception of quality physical education (QPE) among European professionals. S Afr J Res Sport Phys Educ Recreat. 2019;41:31–49. doi: 10.10520/EJC-14fdd81ac8

[34] Ho WKY, Ahmed MD, Khoo S, Tan CH, Dehkordi MR, Gallardo M, et al. Towards developing and validating Quality Physical Education in schools-The Asian physical education professionals’ voice. PLoS One. 2019;14(8):e0218158. doi: 10.1371/journal.pone.0218158 31369586 PMC6675322

[35] Ho W, Ahmed MdD, D’Amico RL de, Ramos A, Ferreira EL, Ferreira MBR, et al. Measuring the perception of quality physical education in Latin American professionals. Revista Brasileira de Ciências do Esporte. 2018;40(4):361–9. doi: 10.1016/j.rbce.2018.05.006

[36] Houser N, Kriellaars D. “Where was this when I was in Physical Education?” Physical literacy enriched pedagogy in a quality physical education context. Front Sports Act Living. 2023;5:1185680. doi: 10.3389/fspor.2023.1185680 37305659 PMC10249748

[37] Uhlenbrock C, Meier HE. The difficulty of policy transfer in physical education: the failure of UNESCO’s Quality Physical Education in South Africa. Physical Education and Sport Pedagogy. 2021;28(2):139–52. doi: 10.1080/17408989.2021.1958176

[38] Cen Y, Zhang S. Theoretical logic, content dimension and enlightenment of UNESCO’s quality physical education policy. Journal of Shenyang Sport University. 2021;40:21–28+43. (in Chinese).

[39] Wang T, Fang Q, Wang J, He J. A study on the impact of adolescent sports policy configuration path on the development of quality physical education: fuzzy-sets qualitative comparative analysis based on 16 countries of the Maritime Silk Road. J Beijing Sport Univ. 2023;46:73–82. doi: 10.19582/j.cnki.11-3785/g8.2023.02.008 (in Chinese).

[40] Zhang X, Guan Q, Shu W. Evolution characteristics and enlightenment of UNESCO’s quality physical education policy. J Chengdu Sport Univ. 2023;49:97–105. doi: 10.15942/j.jcsu.2023.04.014 (in Chinese).

[41] UNESCO. International charter of physical education, physical activity and sport. 2015 [cited 15 Jan 2023]. Available from: https://unesdoc.unesco.org/ark:/48223/pf0000235409?posInSet=1&queryId=b45b2eff-0ad9-4118-9827-033676cc2b31

[42] Ministry of Education, China. Senior Secondary School Physical Education and Health Curriculum Standards. Beijing: People’s Education Press; 2020. (in Chinese).

[43] Ministry of Education, China. Physical Education and Health Programme Standards for Compulsory Education. 2022 Edition. Beijing: Beijing Normal University Press; 2022. (in Chinese)

[44] General Office of the Central Committee of the Chinese Communist Party, General Office of the State Council of the People’s Republic of China. Opinions on comprehensively strengthening and improving school and university physical education work in the new era. 2020 [cited 7 May 2023]. Available from: https://www.gov.cn/gongbao/content/2020/content_5554511.htm. (in Chinese)

[45] Ministry of Education, China. Guidelines for the Reform of Physical Education and Health Teaching (Trial Version). 2021. Available from: http://www.moe.gov.cn/srcsite/A17/moe_938/s3273/202107/t20210721_545885.html. (in Chinese)

[46] General Administration of Sports, China, Ministry of Education, China, National Development and Reform Commission, China. Notice on Enhancing the Quality of After-School Physical Education and Sports Training Services to Promote the Healthy Development of Primary and Secondary School Students. 2022. Available from: https://www.gov.cn/zhengce/zhengceku/2022-07/06/content_5699551.htm?ivk_sa=1023197a. (in Chinese)

[47] Ministry of Education, China. Ensure the Implementation of the Regulation for One Hour of Daily Sport Activities in Primary and Secondary Schools. 2011. Available from: http://www.moe.gov.cn/srcsite/A17/s7059/201107/t20110708_171747.html. (in Chinese)

[48] Ministry of Education, China, Central Propaganda Department, Central Internet Information Office, Central Civilization Office, Ministry of Public Security, Ministry of Civil Affairs, et al. Opinions on Improving the Co-education Mechanism among Schools, Family and Society. 2023 [cited 5 Feb 2023]. Available from: http://www.moe.gov.cn/srcsite/A06/s3325/202301/t20230119_1039746.html. (in Chinese)

[49] Rusticus S. Content validity. In: Maggino F, editor. Encyclopedia of quality of life and well-being research. 2nd edition. Cham: Springer International Publishing; 2023. p. 1384–5.

[50] Tibshirani R, Efron B. An introduction to the bootstrap. Monographs on statistics and applied probability. 1993;57:1–436.

[51] Preacher KJ, Rucker DD, Hayes AF. Addressing Moderated Mediation Hypotheses: Theory, Methods, and Prescriptions. Multivariate Behav Res. 2007;42(1):185–227. doi: 10.1080/00273170701341316 26821081

[52] Bandyrska O, Riznyk V, Yurchak I. Development of electronic engineering design based on non redundant scales theory. 2009 5th International Conference on Perspective Technologies and Methods in MEMS Design. 2009. p. 117–119. Available from: https://ieeexplore.ieee.org/abstract/document/5069726?casa_token=QVoESQpdCM8AAAAA:DbszIDXVyANmMzxJ95IN3NZJB3aHr2lVFTrdKD3Qkbc4YZuB52KWtxWkHOZGdOd0KxX9U5V_3A

[53] Reio TG Jr, Shuck B. Exploratory Factor Analysis. Advances in Developing Human Resources. 2014;17(1):12–25. doi: 10.1177/1523422314559804

[54] Bandalos DL, Finney SJ. Factor analysis: exploratory and confirmatory. 2nd edition. New York: Routledge; 2018.

[55] Kim J-O, Mueller C. Factor analysis: statistical methods and practical issues. SAGE; 1978.

[56] Osborne J. Best practices in quantitative methods. SAGE Publications, Inc.; 2008.

[57] Fu J, Cheng Z, Liu S, Hu Z, Zhong Z, Luo Y. Development and Validation of Peer Relationship Scale for Chinese Community-Dwelling Elderly. Psychol Res Behav Manag. 2021;14:889–903. doi: 10.2147/PRBM.S311352 34234586 PMC8253932

[58] Wang Y, Sun PP. Development and validation of scales for speaking self-efficacy: Constructs, sources, and relations. PLoS One. 2024;19(1):e0297517. doi: 10.1371/journal.pone.0297517 38285668 PMC10824441

[59] Khademi K, Kaveh MH, Asadollahi A, Nazari M. Development and validation of the Women’s Self-care Knowledge and Attitude Questionnaire (WSKAQ). BMC Public Health. 2024;24(1):2338. doi: 10.1186/s12889-024-19831-w 39198800 PMC11360857

[60] Rosenthal MZ, Anand D, Cassiello-Robbins C, Williams ZJ, Guetta RE, Trumbull J, et al. Development and Initial Validation of the Duke Misophonia Questionnaire. Front Psychol. 2021;12:709928. doi: 10.3389/fpsyg.2021.709928 34659024 PMC8511674

[61] Mohammed AH, Ying LH, Boon Hong ML, Sze Nee AW, Ying LS, Ramachandram DS, et al. Development and validation of a knowledge, attitude, and practice (KAP) questionnaire for skin cancer in the general public: KAP-SC-Q. Res Social Adm Pharm. 2024;20(2):124–36. doi: 10.1016/j.sapharm.2023.10.009 37914555

[62] Namdar Areshtanab H, Vahidi M, Hosseinzadeh M, Khani Z. Developing the questionnaire of general population knowledge, attitudes and practices towards the COVID-19 outbreak. Nurs Open. 2024;11(3):e2143. doi: 10.1002/nop2.2143 38520156 PMC10960156

[63] Hair JF, Black WC, Babin BJ, Anderson RE. Multivariate data analysis. 8th edition. Andover: Cengage Learning; 2019.

[64] Mulaik SA, McDonald RP. The Effect of Additional Variables on Factor Indeterminacy in Models with a Single Common Factor. Psychometrika. 1978;43(2):177–92. doi: 10.1007/bf02293861

[65] Tharwat A. Principal component analysis - a tutorial. IJAPR. 2016;3(3):197. doi: 10.1504/ijapr.2016.079733

[66] Meyers LS, Gamst G, Guarino AJ. Applied multivariate research: design and interpretation. 3rd ed. Fargotstein L, McDuffee Y, Weber-Stenis O, West C, editors. USA: Sage publications; 2016.

[67] Costello AB, Osborne J. Best practices in exploratory factor analysis: four recommendations for getting the most from your analysis. Practical Assess Res Eval. 2005;10:1–9. doi: 10.7275/jyj1-4868

[68] Chen X-Q, Jiang X-M, Zheng Q-X, Huang X-X, Liu G-H, Pan Y-Q, et al. Psychometric Properties of the Subhealth Measurement Scale V1.0 for Assessing Suboptimal Health Status of Midwives: A Multicentre Cross-Sectional Study. Perspectives in Psychiatric Care. 2024;2024:1–11. doi: 10.1155/2024/9558391

[69] Jafari A, Nejatian M, Mokhtari AM, Naddafi F, Moshki M. Evaluation the validity and reliability of persian short form of the literacy of suicide scale (LOSS): a methodological study in 2022. BMC Psychiatry. 2023;23(1):783. doi: 10.1186/s12888-023-05281-y 37880611 PMC10601306

[70] Brown TA. Confirmatory Factor Analysis for Applied Research. 2nd edition. Guilford Publications; 2015.

[71] Fornell C, Larcker DF. Evaluating Structural Equation Models with Unobservable Variables and Measurement Error. Journal of Marketing Research. 1981;18(1):39–50. doi: 10.1177/002224378101800104

[72] Marôco J. Análise de Equações Estruturais: Fundamentos teóricos, software & Aplicações. ReportNumber, Lda; 2014.

[73] Cicchetti DV. Guidelines, criteria, and rules of thumb for evaluating normed and standardized assessment instruments in psychology. Psychological Assessment. 1994;6(4):284–90. doi: 10.1037/1040-3590.6.4.284

[74] Pine AE, Baumann MG, Modugno G, Compas BE. Parental involvement in adolescent psychological interventions: A meta-analysis. Clin Child Fam Psychol Rev. 2024;27: 1–20. doi: 10.1007/s10567-024-00481-8 PMC11486598 38748300

[75] Finstad K. Response interpolation and scale sensitivity: evidence against 5-point scales. J Usabil Stud. 2009;5:104–10.

[76] Danielsoper. Free a-priori sample size calculator for structural equation models - free statistics calculators. 2021 [cited 22 Feb 2024]. Available from: https://www.danielsoper.com/statcalc/calculator.aspx?id=89

[77] Kline R. Principles and practice of structural equation modeling. 5th edition. New York: Guilford Publications; 2023.

[78] He Y, Zhou L, Liang W, Liu Q, Liu W, Wang S. Individual, family, and environmental correlates of fundamental motor skills among school-aged children: a cross-sectional study in China. BMC Public Health. 2024;24(1):208. doi: 10.1186/s12889-024-17728-2 38233777 PMC10795326

[79] Li W, Rukavina P. Including overweight or obese students in physical education: a social ecological constraint model. Res Q Exerc Sport. 2012;83(4):570–8. doi: 10.1080/02701367.2012.10599254 23367820

[80] McLeroy KR, Bibeau D, Steckler A, Glanz K. An ecological perspective on health promotion programs. Health Educ Q. 1988;15(4):351–77. doi: 10.1177/109019818801500401 3068205

[81] Matos R, Monteiro D, Amaro N, Antunes R, Coelho L, Mendes D, et al. Parents’ and Children’s (6-12 Years Old) Physical Activity Association: A Systematic Review from 2001 to 2020. Int J Environ Res Public Health. 2021;18(23):12651. doi: 10.3390/ijerph182312651 34886372 PMC8656881

[82] Kingston Ú, Adamakis M, Lester D, Costa J. A Scoping Review on Quality Physical Education Programmes and Their Outcomes on Primary-Level Pupils. Int J Environ Res Public Health. 2023;20(4):3575. doi: 10.3390/ijerph20043575 36834274 PMC9965463

[83] Rocliffe P, O’ Keeffe BT, Sherwin I, Mannix-McNamara P, MacDonncha C. School-based physical education, physical activity and sports provision: A concept mapping framework for evaluation. PLoS One. 2023;18(6):e0287505. doi: 10.1371/journal.pone.0287505 37352181 PMC10289340

[84] Dewi C, Windoro D, Pura DN. Management of Physical Education Facilities and Infrastructure. JET. 2021;5(2). doi: 10.23887/jet.v5i2.34450

[85] Dutrisac S, Bearden AG, Borgel J, Weddell R, Jones M, Oddie S. A tailored physical education program enhances elementary students’ self‐efficacy, attitudes, and motivation to engage in physical activity. Psychology in the Schools. 2023;60(9):3419–34. doi: 10.1002/pits.22927

[86] Guo Q, Samsudin S, Yang X, Gao J, Ramlan MA, Abdullah B, et al. Relationship between Perceived Teacher Support and Student Engagement in Physical Education: A Systematic Review. Sustainability. 2023;15(7):6039. doi: 10.3390/su15076039

[87] Neil-Sztramko SE, Caldwell H, Dobbins M. School‐based physical activity programs for promoting physical activity and fitness in children and adolescents aged 6 to 18 - Neil-Sztramko, SE - 2021 | Cochrane Library. Cochrane Database Syst Rev. 2021;9(9):CD007651. doi: 10.1002/14651858.CD007651.pub3 34555181 PMC8459921

[88] General Office of the Central Committee of the Chinese Communist Party, General Office of the State Council of the People’s Republic of China. Opinions on further reducing the burdens of homework and off-campus training for students during the period of compulsory education. 2021 [cited 12 May 2024]. Available from: https://www.gov.cn/zhengce/2021-07/24/content_5627132.htm (in Chinese).

[89] Epstein J. School, family, and community partnerships: preparing educators and improving schools. Second Edition. New York: Routledge; 2019.

[90] Wang J, Wu S, Chen X, Xu B, Wang J, Yang Y, et al. Impact of awareness of sports policies, school, family, and community environmental on physical activity and fitness among children and adolescents: a structural equation modeling study. BMC Public Health. 2024;24(1):2298. doi: 10.1186/s12889-024-19795-x 39256716 PMC11389504

[91] McCormack M, Pratt M, Conway TL, Cain KL, Frank LD, Saelens BE, et al. Availability of Recreation Facilities and Parks In Relation to Adolescent Participation in Organized Sports and Activity Programs. J Healthy Eat Act Living. 2023;3(1):19–35. doi: 10.51250/jheal.v3i1.59 37794920 PMC10546936

[92] Kellstedt DK, Schenkelberg MA, Essay AM, Von Seggern MJ, Rosenkranz RR, Welk GJ, et al. Youth sport participation and physical activity in rural communities. Arch Public Health. 2021;79(1):46. doi: 10.1186/s13690-021-00570-y 33832548 PMC8028731

