# Supplementary material for: Development and validation of a measurement instrument for student assessment of quality physical education in Chinese secondary schools
Source: PLoS One. 2025 Jun 5;20(6):e0324227. doi: 10.1371/journal.pone.0324227 (PMC12140257; doi:10.1371/journal.pone.0324227)
Supplement: S2 Table — (DOCX) [file pone.0324227.s002.docx]

| **S2 Table.** **Conceptualisation results of 216 label statements** | |
| --- | --- |
| **Conceptualisation** | **Tagged labels** |
| PE teacher-led participation and role modelling in PE | PE teachers should accompany and actively partake alongside students in physical activities and athletic competitions during PE. |
|  | A positive aspect of QPE is that teachers take the initiative to engage in PA, thus acting as role models. |
|  | PE teachers should lead students in physical exercises in PE class. |
|  | PE teachers should engage in PA with students in the class. |
| Cultivating students’ lifelong awareness of PA | QPE should cultivate students’ awareness of the need to participate actively in PA. |
|  | QPE should instil in students the idea that participating in PA is a lifelong commitment. |
| Proper teaching and guidance practices of PE teachers | After class, if students ask the PE teacher about areas they need to improve in sports, teachers should provide detailed coaching. |
|  | If some students are not paying attention during PE, the PE teacher gently guides them to refocus. |
|  | PE teachers should point out (students’) shortcomings in motor skills and patiently guide us in practice. |
| Enhancing students’ critical thinking | QPE should cultivate students’ problem-solving abilities. |
| Parental involvement in physical activities | Parents should regularly participate in sports activities with their children on weekends, fostering an active lifestyle. |
|  | It is good for QPE that parents frequently initiate after-school exercises with their children. |
|  | Parents' involvement in PA boosts students’ engagement in PE classes. |
|  | Parents engage in various activities with their children, such as badminton, cricket, and occasional skiing or skating, which positively correlates with students' outcomes in PE. |
| Maintaining sports facilities and equipment in schools | Our school rarely replaces or maintains sporting equipment. |
|  | The sports facilities and equipment at our school are old and worn out. |
| Enhancing students’ psychological benefits | When students participate in high-quality PE, they tend to forget about unpleasant matters. |
|  | During QPE, students can release stress and relax through structured PA. |
|  | QPE should cultivate students’ ability to endure hardships. |
|  | QPE should enhance students’ psychological resilience and well-being. |
| Integrating PE teaching content with students’ daily lives | After school, students and their neighbourhood companions independently organise sports competitions in the community and participate in physical activities after school, applying knowledge from QPE teaching content. |
|  | It is essential for students in QPE to learn and apply sports techniques that are relevant to their daily lives. |
|  | QPE content should incorporate activities relevant to students’ daily lives. |
| Enhancing students’ enjoyable experiences in PE | QPE should have a dual objective of imparting learning and providing enjoyment to students. |
|  | QPE should aim to provide students with a pleasurable experience. |
|  | Secondary schools’ focus on physical attainment and sport-specific skills over students’ enjoyable experiences in PE is imbalanced. |
|  | QPE should be structured in a way that students find them enjoyable. |
|  | The primary objective of PE classes should be to ensure that students derive pleasure from participation. |
|  | QPE should serve as an outlet for students to express themselves freely, deriving pleasure and enjoyment. |
|  | Students must perceive QPE as a source of enjoyment. |
| Efficient school PA routines | Students feel the PA during school recess is monotonous and boring. |
|  | School recess physical activities should be a fun and active experience for students. |
|  | Schools should utilise recess time to conduct various types of PA. |
| Adapting PE to local contexts and available resources | PE teachers should devise a QPE program that is congruent with the local context and needs. |
|  | PE teachers should adapt their teaching methods to align with local sports facilities and equipment availability. |
|  | Schools need to develop their own QPE program based on their conditions. |
| Creating an inclusive and equality learning environment in PE | PE teachers should foster an inclusive atmosphere in PE class. |
|  | The teaching content of QPE is designed to serve every student's needs. |
|  | There are students with superior physical fitness and those with inferior physical fitness; a balance should be achieved in the PE class. |
|  | PE teachers should provide support and assistance to every student. |
|  | PE teachers should adopt different teaching methods for different students. |
|  | In PE, each student garners the attention of the PE teacher. |
| Diversifying and enriching PE content | QPE teaching content needs enrichment. |
|  | Junior secondary school PE teaching content should not be exclusively focused on national physical examination. |
|  | QPE content should include diverse PA. |
|  | QPE teaching content should include sports that keep up with trends. |
|  | Schools should offer QPE teaching content with different sports options, and students should be able to choose classes based on their interests. |
|  | QPE is challenged by the universalisation and homogenisation of its content. |
|  | QPE should offer a diverse range of teaching content that covers essential physical skills and knowledge and engages students actively. |
|  | Incorporating entertaining physical exercises into the teaching content can make PE classes more enjoyable for students. |
|  | QPE teaching content should include more team sports and group activities. |
|  | A variety of competitions and matches should be integrated into QPE curriculum content. |
|  | The most difficult aspect of junior secondary school PE is preparing for entrance exams, which tends to make teaching content tedious. |
|  | The content of QPE should not be exam-oriented and should include a wide range of sports. |
| Engaging students actively in PE | QPE program should ensure that the content is engaging for every student. |
|  | PE teachers should engage students and motivate them to participate in PA actively. |
|  | Students incorporate activities like tug-of-war, basketball, and football in PE. |
|  | Introducing elements of fun into PE can enhance student engagement. |
| Developing students’ physical fitness | The primary objective of junior-level QPE is to ensure that students meet or exceed the National Student Fitness Test. |
|  | QPE should enable students to exercise for physical strengthening. |
|  | Achieving good physical fitness and health is a key goal for students engaged in QPE. |
|  | The most important thing is for students to improve their physical fitness through QPE. |
|  | QPE should ensure the development of students’ physical fitness. |
|  | The primary task of QPE should be to enable each student to achieve the goal of physical exercise. |
|  | The fundamental function of QPE should be physical strengthening. |
|  | At the junior school level, QPE should incorporate physical fitness training as a core component. |
|  | QPE encompasses exercises that enhance arm strength, core strength, and stamina training. |
| Enhancing PE teachers’ professional skills | PE teachers should know how to schedule the appropriate training and what content to teach at different times. |
|  | If the PE teacher lacks professionalism, students may question their teaching ability and the quality of instruction. |
|  | PE teachers should know how to teach children to maximise learning outcomes. |
|  | PE teachers ought to employ their professionalism to effectively engage students in physical activities within the constraints of a limited time allocation. |
|  | Professionalism is the quintessential quality required for a PE teacher. |
| Building students’ confidence | QPE should build students’ confidence through physical activities. |
|  | QPE is not only about learning sports/motor skills but also about building students’ confidence. |
|  | QPE should develop students’ confidence and demonstrate their motor abilities. |
|  | Students can learn, master and effectively use motor skills in PE, building confidence through this process. |
| Cultivating students’ interests and exercise habits | QPE should encourage students who are not fond of sports to engage in PA actively. |
|  | QPE should primarily aim to cultivate students’ interest in one or two sports events that are beneficial for lifelong well-being and align with their preferences. |
|  | Incorporating interest-based exercises in QPE classes caters to students’ preferences and enhances enjoyment. |
|  | QPE should foster a passion for PA for students. |
|  | Through what students learned in PE, they developed a consistent exercise habit. |
|  | QPE should cultivate students’ interest in sports. |
| Aligning PE teaching content with student developmental stages | Currently, PE’s teaching content is challenging to connect well to the level of school-aged students. |
|  | Different grade levels should teach different PE content while emphasising their connections, providing students with fresh experiences at each stage of their education. |
| Parents guiding and supporting students’ PE and PA | Parents encourage students to attend after-school sports training, which helps them develop new athletic skills and strengthen physical exercise. |
|  | Parental supervision, parental expectations for students’ PE outcomes, and students’ sports achievement are related. |
|  | During holidays, students’ parents should supervise their motor skills practice. |
|  | Parental guidance in students’ PA is crucial in QPE. |
|  | Parents facilitate the enrolment of students in extracurricular sports training programs to bolster their proficiency in athletic pursuits. |
|  | Some parents think it is not a big deal during senior high school if their children fail in PE. This attitude can lead to several negative outcomes for their children. |
|  | Some parents will sacrifice students’ study time to allocate more time for PA. |
|  | Parents need to pay attention to PE and the physical development of their children. |
|  | Some parents actively encourage their children to participate in weekend physical activities, such as hiking and climbing. |
|  | If students spend excessive time on homework or watching TV, their parents should encourage them to engage in PA. |
|  | Parents should place greater emphasis on their children's performance in PE. |
|  | Parents should support PE, recognising that physical exercise benefits their children's health and well-being. |
|  | Parents need to create opportunities for students to develop their sports skills after school. |
|  | Students' parents enrolled their children in an after-school sports class. |
|  | Parents should actively guide and encourage their children to participate in physical activities on weekends. |
|  | Some parents are willing to invest in their children’s extracurricular sports training. |
|  | PE performance is proportional to how parents lead their children to participate in PA. |
| Improving school-family communication in PE class | There is a lack of an effective communication mechanism between the school and family in QPE. |
| Increasing in-service training opportunities for PE teachers | If PE teachers are not provided ongoing training by their schools, teaching is severely constrained in QPE. |
|  | PE teachers lacking essential training are unlikely to provide high-quality instruction. |
|  | PE teachers in our school rarely have opportunities for in-service education. |
|  | As PE teachers, we also want the opportunity to attend district and city-level in-serve training, but they are scarce. |
|  | There should be more opportunities for PE teachers to engage in in-service training. |
| Broadening PE assessment methods | The content of PE exams should be more diversified. |
|  | There is a limitation that PE course examinations only follow the entrance exam standards for junior secondary school students. |
|  | It should not be imperative for the content of junior secondary school PE to be solely centred around examination preparation. |
|  | There should be an augmented emphasis on assessing attitudes and learning processes within QPE. |
| School leadership committed to QPE implementation | Strong school leadership support is crucial for implementing a thorough QPE program. |
|  | The commitment of school leadership to QPE boosts the efficiency of PE teachers in their work. |
|  | High-quality PE hinges on school leadership’s commitment. |
|  | Our school leader exhibits a lack of emphasis on PE. |
|  | Our school leaders tend to prioritise students’ academic achievements over QPE. |
| Fostering positive relationships between PE teachers and students | PE teachers should be able to form positive relationships with their students. |
|  | As a PE teacher, I aim to ensure that students feel comfortable and not intimidated in my presence. |
|  | PE teachers should be gentle and kind in PE class. |
|  | PE teachers should respect students’ ideas. |
|  | Students will feel that interacting with the PE teacher does not impose any pressure. |
|  | There should be a strong sense of affinity between PE teachers and students. |
|  | PE teachers should be very approachable and amiable. |
|  | Students can often feel their relationship with PE teachers is as close and warm. |
| Improving availability of sports equipment and facilities in school | In PE, students often face prolonged waiting times for practice, with their turns being notably brief due to a shortage of sports equipment. |
|  | Schools in rural regions have recently seen improvements in the availability of their sports facilities and equipment. |
|  | High student-to-facility ratios lead to overcrowding in PE classes, which can negatively impact QPE and the overall student experience. |
|  | QPE requires adequate facilities and equipment. |
|  | Schools must prioritise sufficient sports equipment and play space. |
|  | Some schools lack sports equipment. |
|  | Schools often face limited availability of sports facilities. |
|  | In the main metropolis, schools lack land for sports facilities. |
|  | No indoor arenas are at the school. |
| Cultivating students’ social skills | QPE should teach students valuable qualities such as mutual aid and teamwork. |
|  | QPE should enhance students’ sense of unity and collaboration with others. |
|  | Through group sports activities in QPE, students can understand the importance of unity and cooperation. |
|  | In QPE, students can simulate social roles by participating in sports and competitions to enhance their social adaptability. |
| Organising diverse extracurricular sports activities in school | Schools should organise a variety of game activities. |
|  | Our school organises sports leagues, such as football and basketball, which last several weeks. |
|  | Schools should consider incorporating more basketball, football, and track and field events into their semester schedules. |
|  | Schools should ensure that various PA are carried out every semester. |
|  | The school should organise morning exercises for students. |
|  | Schools should consider adding more ball games every semester. |
|  | Our school frequently arranges basketball matches, badminton and football games. |
|  | The school regularly conducts fun sports activities like relay races and tug-of-war. |
| Imparting health-related knowledge for students | QPE should equip students with the knowledge requisite for maintaining a healthy lifestyle. |
|  | Students can learn about PA-related knowledge in QPE. |
| Establishing PE teachers’ responsibility awareness | If the PE teacher is not responsible, students have to engage in unstructured PA independently in PE class. |
|  | PE teachers should fulfil their duties responsibly. |
|  | PE teachers should be highly responsible. |
|  | Conducting a quality PE requires a responsible teacher. |
| Providing extracurricular sports training services in schools | Our school offers extracurricular sports training services. |
|  | Schools should implement multi-dimensional PE in QPE programs, such as offering after-school extracurricular sports training services. |
|  | Our school offers extracurricular sports training services, allowing students to select programs based on their interests and preferences. |
| Conducting PE focus group meetings for PE teachers | In our school, the PE subject has its own research and teaching group that regularly holds PE focus group meetings. |
|  | Our school regularly conducts PE focus meetings for PE teachers to address issues in PE. |
|  | During PE focus group meetings, PE teachers actively discuss teaching issues to improve the quality of PE. |
|  | PE teachers could improve the quality of teaching through PE focus group meetings. |
| Supporting from non-PE teachers and peers on QPE | The level of emphasis non-PE teachers and peers place on PE is very important for PE development. |
|  | Other subject teachers at our school support students participating in PE and PA. |
| Focusing on developing students’ sport-specific skills | QPE should emphasise developing specialised skills in sports like basketball and football. |
|  | Secondary school students should improve their sports expertise through QPE. |
|  | Secondary-level education must focus on teaching sport-specific skills, strategies, and tactics. |
|  | Fostering sports specialisation should be an integral component of PE in secondary schools. |
|  | Secondary-level PE should target sport-specific skills in students’ preferred sports. |
|  | Secondary schools need to direct their efforts toward cultivating specialised skills for students in sports. |
| Promoting community-based sports training services | As part of QPE, students should be encouraged to enrol in sports training classes within their communities after school. |
|  | Sports training institutions in the students’ community make it convenient to participate in physical exercise after school. |
| Emphasising government policy support for QPE | The government's policy attention towards PE in secondary schools is somewhat lacking. |
|  | Government policy is very important for QPE and students’ physical development. |
|  | Government policy tends to support academic subjects, such as Chinese and mathematics but does not pay much attention to PE. |
|  | In the context of China, government policy for PE is the most important. |
| Enhancing the modernisation of sports facilities and equipment in schools | Schools need strategic planning to build and modernise sports facilities and equipment. |
|  | The school’s sports facilities and equipment should be more modernised. |
|  | Schools need ample equipment and modern infrastructure for PE. |
| Structuring PE classes effectively | PE class should be effective by facilitating student assembly and warm-up exercises and then allocating time for main teaching contents. |
|  | Currently, our school has a loose structure for PE classes. |
|  | The structure of PE in senior secondary schools tends to be somewhat loosely organised. |
|  | The PE schedule is not very systematic. |
| Facilitating access to sports equipment in school | Students can easily borrow sports equipment from school after class. |
|  | Some schools place sports equipment near the playground, allowing students to access it easily during recess or after class. |
| Creating a positive classroom atmosphere in PE class | The atmosphere in PE class is important, and a positive environment is conducive to learning |
|  | Students can feel the atmosphere in PE class to be relaxed, which brings them joy. |
|  | The atmosphere in QPE classes is often more relaxed than in other subjects. |
|  | A PE teacher’s sense of humour significantly creates a positive class atmosphere. |
|  | A positive atmosphere in PE classes contributes to an enjoyable experience for students. |
| Increasing government funding for PE | The funding allocated by the government for PE and sports development is insufficient. |
|  | The quality of a school’s PE depends on the grants provided by the government for PE development. |
|  | Schools need to have enough funding from the government for the development of QPE. |
|  | The government’s funding is more likely to allocate education subsidies to core subjects such as Chinese and mathematics, potentially overlooking the importance of PE. |
| Fostering school sports team development | A successful experience of our school’s PE is the breakthrough in sports team training. |
|  | The performance of the school’s sports teams can indicate the school’s PE quality. |
|  | School sports teams are an important component of school PE. |
| Ensuring high qualifications of PE teachers | Our school has high standards for the qualifications of PE teachers. |
|  | Our school’s criteria for recruiting PE teachers is to have a master’s degree, be a national-level athlete, and have been successful in athletic events. |
|  | High-quality PE teachers are essential for implementing high-quality PE. |
| Providing sports equipment and facilities at home | Parents should buy sports equipment for their children. |
|  | Students’ home environment should be suitable for moderate PA. |
|  | Students should have the usual sports equipment at home for their PA. |
| Organising sports events in the community | Community-organised sports competitions allow student participation and can also help develop students’ sports skills. |
|  | Students’ community organises PA and sports competitions where they can showcase the sports skills they have learned in PE. |
| Promoting student autonomy in PE class | Students enjoy the free activity portion of PE class because they can choose the PA that they like. |
|  | Increasing autonomy in PE classes can lead to a more positive experience for students. |
| Enhancing students’ interpersonal relationships | QPE can strengthen the cohesion of the class as a collective. |
|  | QPE could enhance relationships among students. |
|  | In QPE, students and their classmates participate in PA together, which helps strengthen their friendships. |
| Developing students' fundamental motor skills | QPE should be instrumental in equipping students with basic motor skills. |
|  | QPE should serve as a platform for students to acquire fundamental motor skills. |
| Enhancing sports equipment and facilities in the community | Students can frequently engage in exercise because their community offers a variety of sports facilities. |
|  | Students can frequently play badminton as there is a badminton court in their community. |
|  | Students' communities can provide sports equipment, making it convenient for them to practice after school. |
|  | Communities and public areas should enhance their sports infrastructure to attract students to participate in physical activities. |
|  | Students can frequently participate in sports activities because their community has sports facilities downstairs. |
| Integrating community sports resources into QPE programs | Schools should be able to utilise community sports equipment and facilities for QPE. |
|  | Our school has hired experienced coaches from the community to provide after-school sports training services for students. |
| Promoting students’ holistic development | The ultimate goal of QPE is to cultivate well-rounded individuals. |
