# Supplementary material for: Development and validation of a measurement instrument for student assessment of quality physical education in Chinese secondary schools
Source: PLoS One. 2025 Jun 5;20(6):e0324227. doi: 10.1371/journal.pone.0324227 (PMC12140257; doi:10.1371/journal.pone.0324227)
Supplement: S3 Table — (DOCX) [file pone.0324227.s003.docx]

| **S3 Table.** **Open coding results** | | |
| --- | --- | --- |
| **Categorisations** | **Conceptualisation** | **Label numbers** |
| Students’ development | Cultivating students’ lifelong awareness of PA | 2 |
|  | Enhancing students’ critical thinking | 1 |
|  | Enhancing students’ psychological benefits | 4 |
|  | Developing students’ physical fitness | 9 |
|  | Building students’ confidence | 4 |
|  | Cultivating students' interests and exercise habits | 6 |
|  | Cultivating students’ social skills | 4 |
|  | Imparting health-related knowledge for students | 2 |
|  | Focusing on developing students’ sport-specific skills | 6 |
|  | Enhancing students’ interpersonal relationships | 3 |
|  | Developing students’ fundamental motor skills | 2 |
|  | Promoting students’ holistic development | 1 |
| Students’ engagement and experiences in PE | Enhancing students’ enjoyable experiences in PE | 7 |
|  | Engaging students actively in PE | 4 |
| Parents’ engagement and attitude toward PE and PA | Parental involvement in physical activities | 4 |
|  | Parents guiding and supporting students’ PE and sports | 17 |
| Home-based sports resources | Providing sports equipment and facilities at home | 3 |
| PE teacher | PE teacher-led participation and role modelling in PE | 4 |
|  | Proper teaching and guidance practices of PE teachers | 3 |
|  | Enhancing PE teachers’ professional skills | 5 |
|  | Increasing in-service training opportunities for PE teachers | 5 |
|  | Fostering positive relationships between PE teachers and students | 8 |
|  | Establishing PE teachers’ responsibility awareness | 4 |
|  | Ensuring high qualifications of PE teachers | 3 |
| Sports facilities and equipment in the school | Maintaining sports facilities and equipment in schools | 2 |
|  | Improving the availability of sports equipment and facilities in school | 9 |
|  | Enhancing the development and modernisation of sports facilities and equipment in schools | 3 |
|  | Facilitating access to sports equipment in school | 2 |
| PE curriculum | Integrating PE teaching content with students’ daily Lives | 3 |
|  | Adapting PE to local contexts and available resources | 3 |
|  | Creating an inclusive and equality learning environment in PE | 6 |
|  | Diversifying and enriching PE content | 12 |
|  | Aligning PE teaching content with student developmental stages | 2 |
|  | Broadening PE assessment methods | 4 |
|  | Structuring PE classes effectively | 4 |
|  | Creating a positive classroom atmosphere in PE class | 5 |
|  | Promoting student autonomy in PE class | 2 |
| School-based extracurricular PA programs | Efficient school PA routines | 3 |
|  | Organising diverse extracurricular sports activities in school | 8 |
|  | Providing extracurricular sports training services in schools | 3 |
|  | Fostering school sports team development | 3 |
| Co-operation family-school-community in PE | Improving school-family communication in PE class | 1 |
|  | Integrating community sports resources into QPE programs | 2 |
| School leadership and school community support for PE | School leadership committed to QPE implementation | 5 |
|  | Conducting PE focus group meetings for PE teachers | 4 |
|  | Supporting from non-PE teachers and peers on QPE | 2 |
| Community-based sports engagement | Promoting community-based sports training services | 2 |
|  | Organising sports events in the community | 2 |
|  | Enhancing sports equipment and facilities in the community | 5 |
| Government support for PE | Emphasising government policy support for QPE | 4 |
|  | Increasing government funding for PE | 4 |
