# Supplementary material for: Development and validation of a measurement instrument for student assessment of quality physical education in Chinese secondary schools
Source: PLoS One. 2025 Jun 5;20(6):e0324227. doi: 10.1371/journal.pone.0324227 (PMC12140257; doi:10.1371/journal.pone.0324227)
Supplement: S4 Table — (DOCX) [file pone.0324227.s004.docx]

| **S4 Table. Results of the axis coding for 12 categories** | |
| --- | --- |
| **Subcategories** | **Subsidiary categories** |
| Students level | Students’ development |
|  | Students’ engagement and experiences in PE |
| Family level | Parents’ engagement and attitude toward PE and PA |
|  | Home-based sports resources |
| School level | Sports facilities and equipment in the school |
|  | PE curriculum |
|  | PE teacher |
|  | School-based PA programs |
|  | Co-operation family-school-community in PE |
|  | School leadership and school community support for PE |
| Community level | Community-based sports resources |
| Government level | Government support for PE |
