# Supplementary material for: Development and validation of a measurement instrument for student assessment of quality physical education in Chinese secondary schools
Source: PLoS One. 2025 Jun 5;20(6):e0324227. doi: 10.1371/journal.pone.0324227 (PMC12140257; doi:10.1371/journal.pone.0324227)
Supplement: S5 Table — (DOCX) [file pone.0324227.s005.docx]

| **S5 Table.** **Description of items** | | | | |
| --- | --- | --- | --- | --- |
| **Subscales** | **Dimensions** | **Num.** | **Items** | **Description of items** |
| Student subscale | Students’ development | 1 | STL 1 | PE enables me to acquire fundamental motor skills (e.g., running, jumping, throwing). |
|  |  | 2 | STL 2 | PE equipes me with specialised sports skills, such as those required for football, basketball, and badminton. |
|  |  | 3 | STL 3 | PE helps me maintain both physical and mental well-being. |
|  |  | 4 | STL 4 | PE teaches me sports skills as well as knowledge about healthy living. |
|  |  | 5 | STL 5 | PE helps me develop a positive and healthy mindset and lifestyle habits in daily life. |
|  |  | 6 | STL 6 | PE motivates me to participate regularly in physical exercise after school. |
|  |  | 7 | STL 7 | PE cultivates my interest in sports. |
|  |  | 8 | STL 8 | PE enhances my skills in interacting with others, cooperating within a team, and fostering a team spirit. |
|  |  | 9 | STL 9 | Through PE, I develop problem-solving skills. |
|  | Students’ engagement and experience in PE | 10 | STL 10 | I often have many enjoyable experiences PE. |
|  |  | 11 | STL 11 | PE always make me feel very excited. |
|  |  | 12 | STL 12 | I enjoy PE because it is fun. |
|  |  | 13 | STL 13 | I am very focused during PE. |
|  |  | 14 | STL 14 | I look forward to every PE class. |
| Family subscale | Parents’ engagement and attitude in PE and PA | 15 | FL 1 | My parents view PE as highly important. |
|  |  | 16 | FL 2 | My parents always encourage me to actively participate in PE classes and extracurricular PA. |
|  |  | 17 | FL 3 | My parents always acknowledge the progress I make in PE classes. |
|  |  | 18 | FL 4 | My parents frequently guide me in developing my sports skills. |
|  |  | 19 | FL 5 | My parents often accompany me in participating in physical activities. |
|  |  | 20 | FL 6 | My parents provide sports equipment for me. |
|  |  | 21 | FL 7 | My parents purchase sports training courses that align with my interests. |
|  | Home-based sports resources | 22 | FL 8 | My home is outfitted with appropriate and secure sports facilities and equipment. |
|  |  | 23 | FL 9 | My home features sufficient and secure areas for PA. |
|  |  | 24 | FL 10 | My family frequently maintains and updates sports facilities and equipment. |
|  |  | 25 | FL 11 | I reside with their parents (mother and father). |
|  |  | 26 | FL 12 | My family environment is characterised by harmony and warmth. |
| School subscale | Sports facilities and equipment in the school | 27 | SCL 1 | The school is equipped with an adequate range of sports equipment and facilities. |
|  |  | 28 | SCL 2 | The sports equipment and facilities at schools are frequently updated and maintained. |
|  |  | 29 | SCL 3 | I have convenient access to the school’s sports equipment and facilities, particularly after classes. |
|  | PE curriculum | 30 | SCL 4 | The PE program considers the local climate, environment, and facility conditions. |
|  |  | 31 | SCL 5 | PE curriculum is student-centred. |
|  |  | 32 | SCL 6 | A variety of motor skills and health knowledge form the learning content of PE classes. |
|  |  | 33 | SCL 7 | The PE prioritises a multifaceted assessment approach, focusing on physical and mental development through varied perspectives to effectively evaluate student progress. |
|  |  | 34 | SCL 8 | There are equal opportunities for practice in PE |
|  |  | 35 | SCL 9 | A positive attitude towards PE and the associated values are the focus of assessment in PE classes. |
|  |  | 36 | SCL 10 | The learning content of PE is closely linked to my daily life. |
|  |  | 37 | SCL 11 | I can choose the learning content of PE according to my interests. |
|  |  | 38 | SCL 12 | The PE classes are always well-structured and organised, allowing me to exercise effectively. |
|  |  | 39 | SCL 13 | Efficient organization in PE classes facilitates active participation, leading me to exercise. |
|  | PE teacher | 40 | SCL 14 | The PE teacher treats every student equally in class. |
|  |  | 41 | SCL 15 | The PE teacher is dedicated and responsible, teaching with patience and attention to detail. |
|  |  | 42 | SCL 16 | The PE teacher accurately demonstrates motor and sports techniques and possesses strong professional skills. |
|  |  | 43 | SCL 17 | The PE teacher often leads me to participate together in PA during class. |
|  |  | 44 | SCL 18 | I am able to interact well with my PE teacher, fostering a positive relationship. |
|  | School-based extracurricular PA program | 45 | SCL 19 | The school regularly organises sports festival days. |
|  |  | 46 | SCL 20 | The school has established sports teams, clubs, and training services to conduct extracurricular sports activities actively. |
|  |  | 47 | SCL 21 | On days without PE classes, the school arranges for us to participate in one hour of physical exercise in the afternoon after classes. |
|  |  | 48 | SCL 22 | The school organises well-structured and effective physical exercises during major recess every day. |
|  | School leadership and school community support for PE | 49 | SCL 23 | Other teachers, such as class teachers, actively support us in participating in sports activities and PE classes. |
|  |  | 50 | SCL 24 | My friends are actively involved in sports activities and PE and encourage me to be active in sports activities and PE classes. |
|  |  | 51 | SCL 25 | The school consistently conducts three weekly PE classes (two for senior high school), rarely disrupted by other courses or activities. |
|  | Co-operation family-school-community in PE | 52 | SCL 26 | The school regularly hosts school-family collaborative sports events and fun sports days. |
|  |  | 53 | SCL 27 | The school leverages the expertise of excellent coaches from community clubs and training institutions for PE and extracurricular training. |
|  |  | 54 | SCL 28 | Schools often make use of sports grounds, equipment and facilities in the community around the school for PE programmes. |
| Community subscale | Community-based sports resouces | 55 | CL 1 | My community or nearby areas are equipped with safe and appropriate sports equipment and facilities. |
|  |  | 56 | CL 2 | I can conveniently use sports fields, equipment, and facilities in or near their community. |
|  |  | 57 | CL 3 | There are fitness devices suitable for children’s or adolescents’ sports in or near my community. |
|  |  | 58 | CL 4 | There are sports training institutions in or near my community. |
|  |  | 59 | CL 5 | Sports competitions are frequently held in my community. |
|  |  | 60 | CL 6 | Various sports and health knowledge promotion events are regularly organised in my community. |

Note: STL = Student level, FL = Family level, SCL = school level, CL = community level
