# Supplementary material for: Development and validation of a measurement instrument for student assessment of quality physical education in Chinese secondary schools
Source: PLoS One. 2025 Jun 5;20(6):e0324227. doi: 10.1371/journal.pone.0324227 (PMC12140257; doi:10.1371/journal.pone.0324227)
Supplement: S6 Table — (DOCX) [file pone.0324227.s006.docx]

| **S6 Table. Assessment of the items’ mean values** | | | | | |
| --- | --- | --- | --- | --- | --- |
| **Num. of items** | **Rating results** | | | **Mean** | **Experts’ comments** |
|  | **Expert 1** | **Expert 2** | **Expert 3** |  |  |
| STL 1 | 5 | 4 | 5 | 4.7 |  |
| STL 2 | 4 | 4 | 4 | 4.0 |  |
| STL 3 | 5 | 5 | 5 | 5.0 |  |
| STL 4 | 4 | 4 | 4 | 4.0 |  |
| STL 5 | 4 | 4 | 4 | 4.0 |  |
| STL 6 | 5 | 5 | 5 | 5.0 |  |
| STL 7 | 4 | 5 | 4 | 4.3 |  |
| STL 8 | 4 | 4 | 4 | 4.0 |  |
| STL 9 | 5 | 5 | 4 | 4.7 |  |
| STL 10 | 5 | 5 | 4 | 4.7 |  |
| STL 11 | 5 | 4 | 3 | 4.0 |  |
| STL 12 | 5 | 4 | 3 | 4.0 |  |
| STL 13 | 4 | 4 | 4 | 4.0 |  |
| STL 14 | 4 | 4 | 4 | 4.0 |  |
| FL 1 | 5 | 5 | 5 | 5.0 |  |
| FL 2 | 5 | 3 | 3 | 3.7 |  |
| FL 3 | 4 | 5 | 4 | 4.3 |  |
| FL 4 | 4 | 4 | 4 | 4.0 |  |
| FL 5 | 4 | 5 | 4 | 4.3 |  |
| FL 6 | 5 | 3 | 4 | 4.0 |  |
| FL 7 | 3 | 4 | 4 | 3.6 |  |
| FL 8 | 4 | 5 | 4 | 4.3 |  |
| FL 9 | 5 | 5 | 3 | 4.3 |  |
| FL 10 | 5 | 3 | 4 | 4.0 |  |
| FL 11 | 4 | 4 | 4 | 4.0 |  |
| FL 12 | 5 | 4 | 4 | 4.3 |  |
| SCL 1 | 5 | 5 | 5 | 5.0 |  |
| SCL 2 | 4 | 4 | 4 | 4.0 |  |
| SCL 3 | 5 | 4 | 3 | 4.0 |  |
| SCL 4 | 4 | 4 | 4 | 4.0 |  |
| SCL 5 | 5 | 4 | 4 | 4.3 |  |
| SCL 6 | 5 | 4 | 4 | 4.3 |  |
| SCL 7 | 2 | 4 | 3 | 3.0 | Change to ‘The PE class adopts a multifaceted assessment approach to measure my performance’ |
| SCL 8 | 4 | 3 | 3 | 3.3 | Change to ‘There are equal opportunities for all students to practice in PE’ |
| SCL 9 | 5 | 4 | 4 | 4.3 |  |
| SCL 10 | 5 | 3 | 4 | 5.0 |  |
| SCL 11 | 5 | 3 | 3 | 3.7 |  |
| SCL 12 | 3 | 3 | 5 | 3.7 |  |
| SCL 13 | 3 | 2 | 4 | 3.0 | Change to ‘Structured PA during PE classes encourages my active participation, allowing me to engage in exercises that lead to sweating’ |
| SCL 14 | 4 | 4 | 4 | 4.0 |  |
| SCL 15 | 4 | 3 | 4 | 3.7 |  |
| SCL 16 | 4 | 3 | 3 | 3.3 | Change to ‘The PE teacher accurately demonstrates motor and sports techniques and shows strong professional competence’ |
| SCL 17 | 5 | 4 | 4 | 4.3 |  |
| SCL 18 | 5 | 4 | 4 | 4.3 |  |
| SCL 19 | 5 | 5 | 5 | 5.0 |  |
| SCL 20 | 5 | 5 | 5 | 5.0 |  |
| SCL 21 | 5 | 4 | 5 | 4.7 |  |
| SCL 22 | 4 | 4 | 4 | 4.0 |  |
| SCL 23 | 3 | 4 | 3 | 3.0 | ’ |
| SCL 24 | 4 | 4 | 4 | 4.0 |  |
| SCL 25 | 4 | 4 | 3 | 3.7 |  |
| SCL 26 | 3 | 4 | 3 | 3.3 | Change to ‘The school promotes a family-school-community collaborative PE program and organises sports events’ |
| SCL 27 | 4 | 4 | 5 | 4.3 |  |
| SCL 28 | 5 | 4 | 2 | 3.6 | Changed to ‘The school has implemented PE homework supervised by parents at home’ |
| CL 1 | 5 | 4 | 5 | 4.7 |  |
| CL 2 | 4 | 4 | 4 | 4.0 |  |
| CL 3 | 5 | 5 | 5 | 5.0 |  |
| CL 4 | 4 | 4 | 4 | 4.0 |  |
| CL 5 | 5 | 4 | 5 | 4.7 |  |
| CL 6 | 4 | 3 | 4 | 3.7 |  |
| Note: STL = Student level, FL = Family level, SCL = School level, CL = Community level | | | | | |
