# Supplementary material for: Development and validation of a measurement instrument for student assessment of quality physical education in Chinese secondary schools
Source: PLoS One. 2025 Jun 5;20(6):e0324227. doi: 10.1371/journal.pone.0324227 (PMC12140257; doi:10.1371/journal.pone.0324227)
Supplement: S7 Table — (DOCX) [file pone.0324227.s007.docx]

| **S7 Table. Assessment of the items’ content validity index** | | | | |
| --- | --- | --- | --- | --- |
| **Num. of items** | **Rating results** | | | **I-CVI** |
|  | **Expert 4** | **Expert 5** | **Expert 6** |  |
| STL 1 | 4 | 4 | 4 | 1 |
| STL 2 | 4 | 4 | 4 | 1 |
| STL 3 | 4 | 4 | 4 | 1 |
| STL 4 | 4 | 4 | 4 | 1 |
| STL 5 | 4 | 4 | 4 | 1 |
| STL 6 | 4 | 4 | 4 | 1 |
| STL 7 | 4 | 4 | 4 | 1 |
| STL 8 | 4 | 4 | 4 | 1 |
| STL 9 | 3 | 3 | 4 | 1 |
| STL 10 | 4 | 4 | 3 | 1 |
| STL 11 | 4 | 3 | 4 | 1 |
| STL 12 | 4 | 4 | 2 | 0.66 |
| STL 13 | 4 | 4 | 4 | 1 |
| STL 14 | 4 | 3 | 3 | 1 |
| FL 1 | 4 | 4 | 4 | 1 |
| FL 2 | 4 | 4 | 4 | 1 |
| FL 3 | 4 | 4 | 4 | 1 |
| FL 4 | 4 | 4 | 4 | 1 |
| FL 5 | 4 | 4 | 4 | 1 |
| FL 6 | 4 | 4 | 4 | 1 |
| FL 7 | 4 | 4 | 4 | 1 |
| FL 8 | 4 | 3 | 3 | 1 |
| FL 9 | 3 | 4 | 3 | 1 |
| FL 10 | 3 | 3 | 3 | 1 |
| FL 11 | 2 | 2 | 2 | 0 |
| FL 12 | 2 | 2 | 2 | 0 |
| SCL 1 | 4 | 4 | 4 | 1 |
| SCL 2 | 4 | 4 | 4 | 1 |
| SCL 3 | 4 | 4 | 4 | 1 |
| SCL 4 | 4 | 3 | 4 | 1 |
| SCL 5 | 4 | 4 | 4 | 1 |
| SCL 6 | 4 | 4 | 4 | 1 |
| SCL 7 | 3 | 4 | 4 | 1 |
| SCL 8 | 4 | 4 | 4 | 1 |
| SCL 9 | 4 | 3 | 3 | 1 |
| SCL 10 | 4 | 4 | 4 | 1 |
| SCL 11 | 3 | 4 | 3 | 1 |
| SCL 12 | 4 | 4 | 4 | 1 |
| SCL 13 | 4 | 4 | 4 | 1 |
| SCL 14 | 4 | 3 | 2 | 0.66 |
| SCL 15 | 4 | 4 | 4 | 1 |
| SCL 16 | 4 | 4 | 4 | 1 |
| SCL 17 | 4 | 4 | 4 | 1 |
| SCL 18 | 4 | 4 | 4 | 1 |
| SCL 19 | 4 | 4 | 4 | 1 |
| SCL 20 | 4 | 4 | 4 | 1 |
| SCL 21 | 4 | 4 | 4 | 1 |
| SCL 22 | 4 | 4 | 4 | 1 |
| SCL 23 | 4 | 4 | 4 | 1 |
| SCL 24 | 4 | 4 | 4 | 1 |
| SCL 25 | 4 | 3 | 3 | 1 |
| SCL 26 | 4 | 3 | 4 | 1 |
| SCL 27 | 3 | 4 | 4 | 1 |
| SCL 28 | 4 | 4 | 4 | 1 |
| CL 1 | 3 | 4 | 3 | 1 |
| CL 2 | 4 | 3 | 3 | 1 |
| CL 3 | 2 | 3 | 4 | 0.66 |
| CL 4 | 4 | 4 | 4 | 1 |
| CL 5 | 4 | 4 | 4 | 1 |
| CL 6 | 4 | 3 | 3 | 1 |
| Note: STL = Student level, FL = family level, SCL = School level, CL = Community level | | | | |
